# Light triggered nanoscale biolistics for efficient intracellular delivery of functional macromolecules in mammalian cells

**DOI:** 10.1038/s41467-022-29713-7

**Published:** 2022-04-14

**Authors:** Juan C. Fraire, Elnaz Shaabani, Maryam Sharifiaghdam, Matthias Rombaut, Charlotte Hinnekens, Dawei Hua, Jana Ramon, Laurens Raes, Eduardo Bolea-Fernandez, Toon Brans, Frank Vanhaecke, Peter Borghgraef, Chaobo Huang, Félix Sauvage, Tamara Vanhaecke, Joery De Kock, Ranhua Xiong, Stefaan De Smedt, Kevin Braeckmans

**Affiliations:** 1 Laboratory for General Biochemistry and Physical Pharmacy, Faculty of Pharmaceutical Sciences, Ghent University, 9000 Ghent, Belgium; 2 Department of In vitro Toxicology and Dermato-Cosmetology, Faculty of Medicine and Pharmacy, Vrije Universiteit Brussel (VUB), 1090 Brussels, Belgium; 3 Joint Laboratory of Advanced Biomedical Materials (NFU‐UGent), College of Chemical Engineering, Nanjing Forestry University (NFU), 210037 Nanjing, P. R. China; 4 Department of Chemistry, Atomic & Mass Spectrometry – A&MS Research Group, Ghent University, Campus Sterre, Krijgslaan 281-S12, 9000 Ghent, Belgium; 5 VIB Bioimaging Core Ghent, VIB, 9000 Ghent, Belgium

**Keywords:** Biolistics, Transfection, Nanostructures, Nanoparticles, Nanostructures

## Abstract

Biolistic intracellular delivery of functional macromolecules makes use of dense microparticles which are ballistically fired onto cells with a pressurized gun. While it has been used to transfect plant cells, its application to mammalian cells has met with limited success mainly due to high toxicity. Here we present a more refined nanotechnological approach to biolistic delivery with light-triggered self-assembled nanobombs (NBs) that consist of a photothermal core particle surrounded by smaller nanoprojectiles. Upon irradiation with pulsed laser light, fast heating of the core particle results in vapor bubble formation, which propels the nanoprojectiles through the cell membrane of nearby cells. We show successful transfection of both adherent and non-adherent cells with mRNA and pDNA, outperforming electroporation as the most used physical transfection technology by a factor of 5.5–7.6 in transfection yield. With a throughput of 10^4^-10^5^ cells per second, biolistic delivery with NBs offers scalable and highly efficient transfections of mammalian cells.

## Abstract (web summary)

Ballistic delivery with micro/nano-particles has been successfully used to transfect plant cells, however, has failed in mammalian cells due to toxic effects. Here, the authors report on a self-assembled nano-ballistic delivery system for the delivery of functional macromolecules and demonstrate efficient transfection of mammalian cells.

## Introduction

Delivering exogenous compounds into cultured cells in vitro or ex vivo is an ubiquitous requirement in biomedical and biotechnological applications, whether it is for the creation of mutant cell lines in biology, for drug screening of biologicals in pharmacy, or for the production of engineered cells for cell-based therapies. The types of molecules that need to be delivered are very diverse, ranging from small molecules, over peptides and proteins, to different types of nucleic acids. Regardless of the specific application, the common challenge is to deliver those compounds safely and efficiently across the cell membrane^1^. One option is to use commercial transfection reagents (i.e., RNAiMAX or jet-PEI) which usually can achieve high delivery efficiencies with high levels of cell viability, depending on the cell type and cargo to be delivered^1^. However, they are usually optimized to deliver one type of cargo, like nucleic acids, but are less suited to deliver other types of molecules, like proteins. Moreover, they often perform less well on hard-to-transfect cell types, such as T cells which are considered to be profoundly recalcitrant to carrier-mediated transfection due to poor endocytic uptake and inefficient endosomal release^2^. This is why especially in the last decade several types of cell-membrane disruption methods have emerged, like (nanoneedle) electroporation, cell squeezing or photoporation^2–4^. These physically triggered delivery methods not only allow to deliver a broad variety of membrane-impermeable effector molecules into many different cell types, they also are currently the preferred strategy for ex vivo transfection of difficult to transfect cells like immune cells (including T cells, B cells, natural killer cells, dendritic cells, and macrophages), primary stem cells, cells of the hematopoietic lineage, and neurons^4^.

A distinction is made between two types of cell membrane disruption methods^4^, one being based on membrane permeabilization and the other on membrane penetration. Cell membrane permeabilization methods have been explored the most so far, with electroporation being the oldest and best-known to date, which has proven its worth to transfect many different cell types since its conception in the 1980s^5^. However, it is often associated with acute cell death and high levels of cell stress, even leading to alteration of the cell’s homeostasis and normal functioning^6^. Especially in the past decade more gentle permeabilization technologies have been explored thanks to the emergence of nanomaterials and nanofabrication methods. Prominent examples are permeabilization by microfluidic cell squeezing^6–8^ and photoporation (also sometimes referred to as optoporation)^9^.

Methods for direct penetration of the cell membrane are much less explored so far. One prominent example is biolistic intracellular delivery, which employs high-density metal particles (tungsten, gold, or silver) of 0.5–2 µm in size which, accelerated by gunpowder or gas shock waves, collide with target cells and puncture the cell membrane^4,10,11^. However, as it is often associated with excessive cell damage^12,13^, it has not been widely accepted as a standard intracellular delivery technology, even though it has been applied with some success to plants^14^, neurons^15^, and microorganisms^16^. With the emergence of nanotechnology, attempts have been made to refine biolistic delivery further. For example, tubular nanostructures (microcannons) were recently proposed containing a fuel and polystyrene “bullets.” Upon vaporization of the fuel by applying ultrasound energy, the polystyrene bullets are ejected at high velocities, allowing them to penetrate into tissues for drug delivery purposes^17,18^. These tubular microcannons were further developed as “microshotguns” able to cross the tympanic membrane for drug delivery in the middle ear^19^. In spite of these interesting proof-of-concept studies, clinical translation seems challenging given the difficulty to remove the nanostructures after penetration of the cell membrane or to design such systems based on more biodegradable materials.

**Fig. 1:**
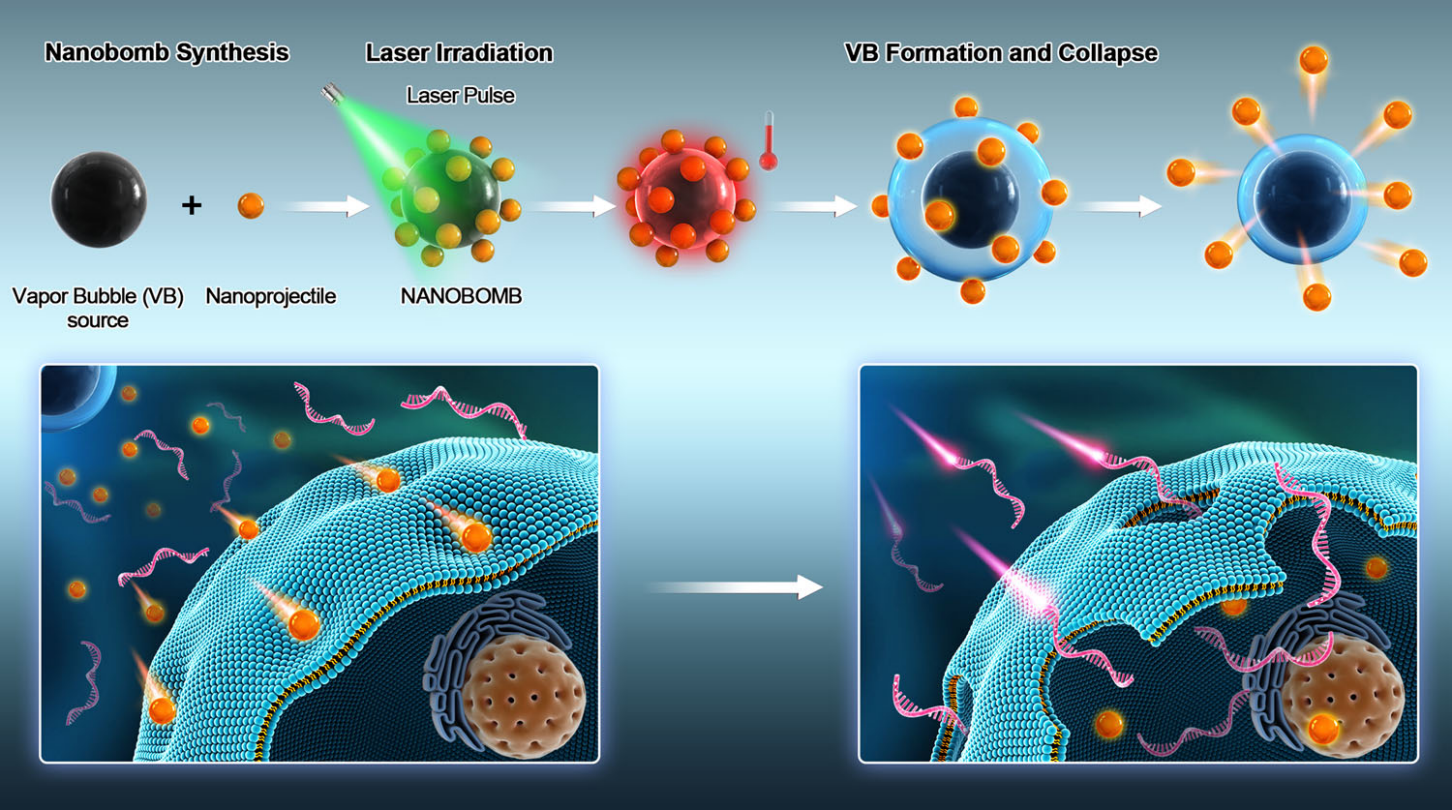
Optically triggered nanobombs are composed of a vapor bubble (VB) core particle to which nanoprojectiles are conjugated. Upon irradiation with an intense laser pulse, the nanobomb core will heat up to several hundreds of degrees Celsius, thus evaporating the surrounding water and forming a VB. The mechanical forces by VB expansion and collapse propel the nanoprojectiles across the plasma membrane of nearby cells. Foreign molecules, such as mRNA, present in the cell medium can then migrate into the cell’s cytoplasm.

In order to develop an effective and widely usable nano-biolistic system, we here propose light-triggered self-assembled “nanobombs” (NBs), designed to eject nanoprojectiles which can penetrate through a cell membrane. As illustrated in Fig. 1, these light-triggered NBs consist of a photothermal core particle coated with smaller nanoparticles that act as “nanoprojectiles.” Upon irradiation with pulsed laser light, the photothermal core particle will quickly heat up and vaporize the surrounding water, resulting in the formation of a water vapor bubble (VB). We hypothesized that the physical forces generated by the fluid phenomena that emerges because of the quick expansion and collapse of VBs will propel the nanoprojectiles through the surrounding medium and through the membrane of nearby cells. This way pores are created in the cell membrane through which exogenous compounds can enter the cell’s cytoplasm. Advantages of this system are facile synthesis, use of biocompatible nanoprojectiles, control of the pore size corresponding to the size of the selected nanoprojectiles, high-throughput treatment of cells, and applicability to both adherent and suspension cells. By choosing nanoprojectiles >100 nm in diameter, pores should be large enough to let even very large nucleic acids such as mRNA and pDNA enter cells.

In this work, we demonstrate successful self-assembly of nanobombs composed of iron-oxide nanoparticles (IONP) as the photothermal core and fluorescent polystyrene nanospheres or biocompatible poly(lactic-co-glycolic acid) (PLGA) nanoparticles as nanoprojectiles. Coupling of the nanoprojectiles to the IONP core particles is achieved through a biotin–streptavidin link. Next, we determine the laser pulse fluence needed for VB generation from the IONP core and investigate release of nanoprojectiles as well as their penetration capabilities using a tissue phantom gel matrix. In a next step, we evaluate the cell-membrane penetration capabilities of light-triggered NBs, demonstrating successful penetration of nanoprojectiles in the cell’s cytoplasm. Successful intracellular delivery is demonstrated first with macromolecular fluorescent model molecules, and subsequently with mRNA and pDNA both in adherent (HeLa) and suspension (Jurkat) cells. Compared to electroporation and photoporation as benchmark technologies, light-triggered NBs yield 5.5–7.6 times more living transfected cells. Finally, we demonstrate that light-triggered NBs can be used for spatially resolved transfections of cells by patterned scanning of the laser beam.

## Results

### Design, characterization, and light-triggered activation of NBs

**Fig. 2:**
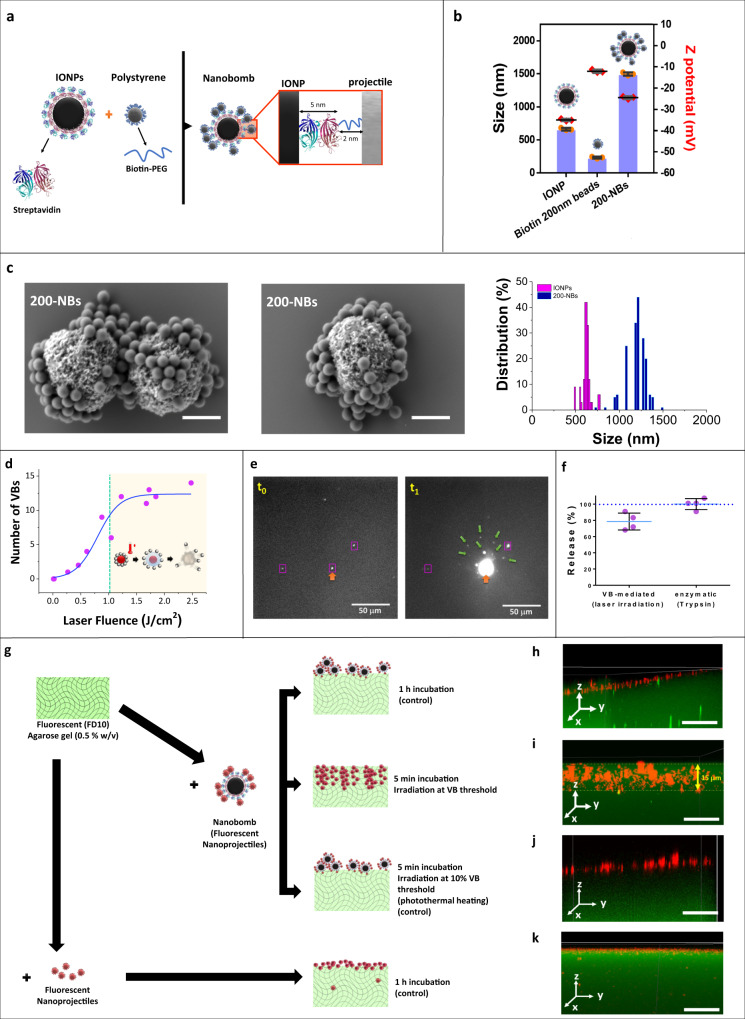
Synthesis, characterization, and laser activation of 200-NBs composed of IONP core particles and 200 nm polystyrene nanospheres as nanoprojectiles. **a** Schematic representation the NB design: polystyrene beads are attached onto IONPs through biotin-STV coupling. **b** DLS size and zeta potential characterization of the different building blocks of 200-NBs (*n* = 3 independent experiments, mean ± SD). **c** Representative SEM images of NBs synthesized using 0.5 µm IONP core particles and 200 nm polystyrene beads with the corresponding size distribution of the 200-NBs and of the IONP core particles. The scale bar represents 500 nm. *n* = 10 images were recorded from two samples. **d** Determination of the vapor bubble (VB) fluence threshold for NBs composed of 0.5 µm IONP core and 200 nm polystyrene beads (200-NBs). The VB regime is indicated in yellow in the graph, to the right-hand side of the dashed green line. **e** Dark field images of nanobombs in water before (*t*_0_) and right after (*t*_1_ = 10 µs) application of a single laser pulse that was directed to the nanobomb indicated by the orange arrow. Nanospheres (green arrows) can be seen to be propelled away from the nanobomb over a distance of a few 10 s of µm. *n* = 10 images were recorded from one sample. **f** Quantification of nanoprojectile release. 200-NBs were activated at the VB threshold fluence (*λ* = 561 nm, 7 ns pulse, 1.22 J/cm^2^), and release of the fluorescent polystyrene nanoprojectiles was quantified based on the fluorescence of the supernatant after magnetic washing. Enzymatic release using 10% trypsin was used as a positive control. n = 3 independent experiments, mean ± SD. **g** Schematic representation of the nanoprojectile penetration in a phantom gel matrix experiment. NBs are incubated with a pre-formed gel either for 1 h without laser irradiation (control) or for 5 min followed by laser irradiation. X-projected 3D confocal image of: **h** the gel incubated for 1 h with 200-NB (1.3 × 10^8^ NBs/mL); **i** gel incubated for 5 min with the same NBs followed by irradiation at the VB threshold; **j** gel incubated for 5 min with the same NBs followed by laser irradiation at 10% of the VB threshold (0.37 J/cm^2^); **k** gel incubated for 1 h with fluorescent polystyrene beads (1% v/v). The scale bar represents 20 μm. For each condition, *n* = 5 images were recorded from two samples.

The NB design consists of a thermal core particle that is surrounded by smaller nanoparticles that act as nanoprojectiles upon light-triggered activation (Fig. 2a). For the NB’s core we used commercially available 0.5 µm magnetic beads, consisting of a polymeric core that is surrounded by a layer of iron-oxide and coated with a covalently attached streptavidin monolayer. We will further refer to these particles as iron oxide nanoparticles (IONP), as the iron oxide shell is the photothermal component needed for VB generation^20^. Biotinylated fluorescent polystyrene nanospheres (100, 200, and 500 nm) were initially selected as nanoprojectiles, as they can be conveniently detected with fluorescence techniques (e.g., confocal microscopy). According to the projectile size, we will refer to those NBs as 100-NBs, 200-NBs, and 500-NBs, respectively. Self-assembly of the NBs was achieved by simply mixing the streptavidin functionalized IONPs with the biotinylated nanospheres (ratio IONP/biotin-nanoprojectiles = 1/1750) overnight under constant stirring, after which they were purified by magnetic isolation. This IONP/nanoprojectile ratio was chosen to have a sufficient excess of 100 nm nanoprojectiles to cover the entire surface of IONP core particles. The same ratio was used for larger nanoprojectile sizes as well for the same reason. Figure 2b shows the DLS characterization of the IONP core particles, 200 nm polystyrene beads and the assembled 200-NBs. A considerable increase in hydrodynamic size was indeed observed upon NB formation. The zeta potential of the 200-NBs became slightly less negative compared to the IONP core particles, which is another indication of the attachment of biotinylated nanoprojectiles. Similar measurements for 100-NBs and 500-NBs are shown in Supplementary Fig. 1a. Figure 2c shows representative SEM images of 200-NBs, further confirming successful NB formation with polystyrene nanospheres being clearly visible around the IONP surface. Image analysis showed an average NB size of 1206 ± 86 nm, quite close to the DLS result. After synthesis the NBs remained stable in PBS buffer for at least three weeks (Supplementary Fig. 1b).

Following successful NB synthesis, we evaluated the formation of laser-induced VB, which is expected to propel the attached nanoprojectiles away from the core particles. VB can be triggered from photothermal nanoparticles upon irradiation with a short laser pulse (typically <10 ns) of sufficient fluence^21^. The VB threshold is commonly defined as the fluence level (J/cm²) at which 90% of the irradiated particles generate a VB. This threshold can be determined experimentally by detecting and quantifying the formation of VBs using dark-field microscopy as a function of the applied laser fluence (Supplementary Fig. 1c)^21,22^. During their lifetime, VB strongly scatter the incident light from the dark-field microscope’s light source, resulting in localized bright flashes of light soon after arrival of the laser pulse. The number of detected VB will gradually increase with increasing laser pulse fluence, until all particles in the irradiated area form VB. This results in the typical sigmoidal curve as shown in Fig. 2d for 200-NBs (7 ns laser pulse, *λ* = 561 nm). After fitting of a Boltzmann curve, the VB threshold was found to be 1.22 J/cm² for the 200-NBs. Similar curves can be found for 100-NBs and 500-NBs in Supplementary Fig. 1di, ii, from which the VB threshold was determined as 1.14 J/cm^2^ and 1.26 J/cm^2^, respectively. The fact that these VB thresholds are similar for all NBs is to be expected since they are all based on the same 0.5 µm photothermal core particle. In addition to ns laser pulses, we also confirmed VB formation upon irradiation with ps laser pulses at the same wavelength (2 ps laser pulse, *λ* = 561 nm). In this case, the VB threshold for 200-NBs was 0.49 J/cm^2^ (Supplementary Fig. 1diii). Not surprisingly, this is lower than for ns laser pulses as ps laser pulses provide more efficient energy transfer into photothermal nanoparticles^23^.

Interestingly, successful propelling of projectiles after NB activation could be visualized by darkfield imaging as well. Figure 2e shows such dark field images of 200-NBs in water before (*t*_0_ = 0 s) and after (*t*_1_ = 10 µs) application of a single laser pulse that was directed to the NB indicated by the orange arrow. Apart from intensely scattered light by the VB, nanoprojectiles (green arrows) can be seen as well as they are propelled away from the nanobomb over a distance of a few 10 s of µm.

SEM images of 100-NBs, 200-NBs, and 500-NBs before and after laser irradiation at the VB threshold are shown in Supplementary Fig. 2a. After laser irradiation, the IONP core particles are found to be highly deformed, likely as a consequence of the high temperatures that are reached within the particles upon laser irradiation in combination with the mechanical stress induced by VB expansion and collapse. The nanoprojectiles on the other hand seem to remain mostly intact.

To quantify the degree of nanoprojectile release, 200-NBs were irradiated (7 ns, *λ* = 561 nm, 1.22 J/cm^2^), and the released beads were separated from the IONP core particles by a magnetic wash. The percentage of released nanoprojectiles was quantified by fluorimetry based on a previously determined calibration curve (Supplementary Fig. 1bi). As a positive control, we included samples incubated with 10% trypsin, which is an enzyme able to degrade streptavidin and is expected to release all nanoprojectiles. As can be seen in Fig. 2f, almost 80% of the nanoprojectiles were successfully released upon laser activation of the 200-NBs. Similar results were obtained for 100-NBs and 500-NBs (Supplementary Fig. 1bii–iv).

To evaluate if the propelled nanoprojectiles can penetrate a physical barrier, their penetration was investigated in a tissue phantom gel made of agarose (0.5 % w/v) with mechanical properties comparable to biological tissue^17^. As shown in the schematic representation of Fig. 2g, the gel was fluorescently labeled by incorporation of FITC-Dextran 10 kDa (FD10) into the gel matrix during solidification, allowing visualization by confocal microscopy. As a control, 200-NBs (1.3 × 10^8^ NPs/mL) were added to the solidified gel and incubated for 1 h, after which they remained mostly on top of the gel (Fig. 2h). Next, when activating NBs after just 5 min incubation by irradiation at the VB threshold (Fig. 2i), nanoprojectiles penetrated up to 15 µm into the gel matrix with an average penetration distance of 8 ± 3 µm. Since there is virtually no heat transfer into the surrounding upon VB formation^1,2^, this observation cannot be attributed to mere heating and melting of the agarose gel. To verify this more explicitly, we proceeded to irradiate the NBs after 5 min incubation at a laser pulse fluence of 10% of the VB threshold (0.37 J/cm^2^). In that case almost no VB can be formed and all heat from the NBs will diffuse into the gel. As can be seen in Fig. 2j, the fluorescent nanobeads attached to the surface of NBs did not penetrate into the gel, providing further confirmation that bead penetration as observed in Fig. 2i is a consequence of the mechanical forces induced by VBs and not just because of thermal heating and melting of the agarose gel. In addition, to verify that penetration of beads was not merely due to passive diffusion of released nanoprojectiles into the gel, 200 nm biotinylated beads (1% v/v) were incubated for 1 h on the gel. Since they remained at the gel surface (Fig. 2k) this confirms that penetration into the gel must have happened by an active force exerted by the light-triggered NBs. The experiment was repeated for 500-NBs as well, with virtually identical results (Supplementary Fig. 2d). Also beads released from 100-NBs were found to penetrate into the gel upon VB generation (Supplementary Fig. 1ci), although in this case results are confounded by the fact that those small nanoparticles could diffuse passively into the gel as well (Supplementary Fig. 2cii). Nevertheless, together these results confirm the ability of light-triggered NBs to propel their nanoprojectiles across a physical barrier.

### Cell membrane penetration by light-triggered NBs

**Fig. 3:**
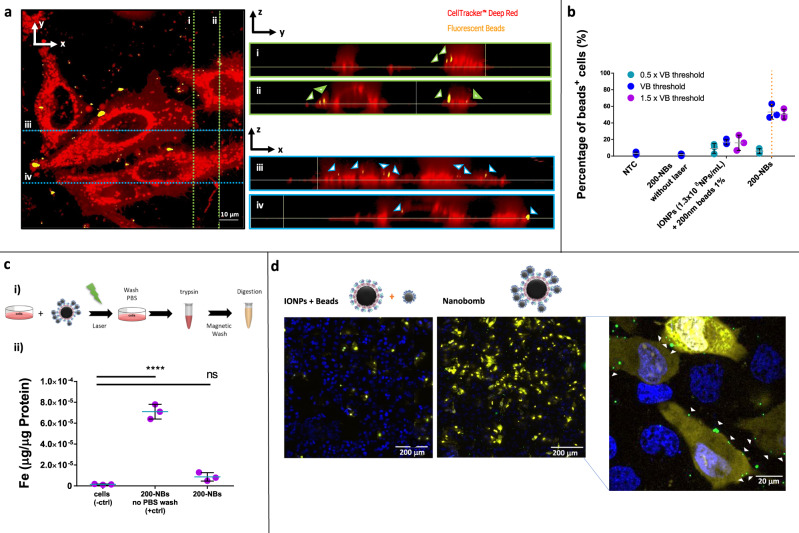
Experimental and theoretical evaluation of 200-NB activation and its interaction with cells. **a** Confocal images showing nanoprojectile penetration (indicated with arrow heads) into the cell’s cytoplasm after activation of 200-NBs. The cytoplasm was stained with CellTracker Deep Red. Different vertical sections are shown as well along the *y* (i and ii) and *x* (iii and iv) direction. *n* = 10 images were recorded from two samples. **b** Flow cytometric quantification of nanoprojectile presence in HeLa cells after 200-NB activation (1.3 × 10^8^ NBs/mL). Samples were irradiated with a laser pulse fluence at 0.5×, 1×, and 1.5× the VB threshold. Controls include 200-NB incubation (1.3 × 10^8^ NBs/mL) without irradiation and the simultaneous incubation of free beads 1% (v/v) and IONPs (1.3 × 10^8^ NPs/mL) with laser irradiation. The percentage of positive cells was quantified by flow cytometry (*n* = 3 biologically independent samples, data presented as mean ± SD). **c** Quantification of iron content in HeLa cells after 200-NB activation. (i): Schematic overview of the experimental procedure to determine the iron content in cells (bulk analysis) by ICP-MS. (ii): The iron concentration was measured in untreated cells (negative control), cells incubated with NBs without washing (positive control), and cells treated by activated 200-NBs and subsequent washing with PBS. The results show the mass of Fe normalized per μg of protein as determined by a BSA protein quantification assay (*n* = 3 biologically independent samples, data presented as mean ± SD, one-way ANOVA, ****P < 0.0001). **d** Laser-triggered NB-mediated delivery of propidium iodide in HeLa cells. With light-triggered nanobombs PI (yellow) could be successfully delivered into most cells, which was not the case in a control experiment where IONP and nanospheres were added as an uncoupled mixture to the cells. For each condition, *n* = 10 images were recorded from two samples. The green fluorescent nanospheres are found inside the cells, leading to pore formation in the cell membrane and the concomitant influx of PI that was added to the cell medium. Cell nuclei stained by Hoechst are visible in blue.

Next, we evaluated to which extent nanoprojectiles ejected from laser-activated NBs can penetrate cell membranes. 200-NBs were added to HeLa cells (1.3 × 10^8^ NBs/mL) and immediately irradiated with laser pulses at the VB threshold. 3D confocal microscopy was used to determine the location of the released nanoprojectiles. For this, we stained the cytoplasm with CellTracker Deep Red and used green fluorescent beads as projectiles. Figure 3a shows an exemplary horizontal confocal section of HeLa cells after activation of the 200-NBs. Different vertical sections are shown as well along the *y* (i and ii) and x (iii and iv) directions. Beads were primarily found in the cytoplasmic stained regions or at the top of the cells, as indicated by the arrows in the z-projections (Fig. 3ai–iv). To study this more quantitatively on a large number of cells, entry of fluorescent nanoprojectiles in cells was evaluated by flow cytometry at different laser pulse fluences (0.5×, 1× and 1.5× VB threshold) for a NB concentration of 1.3 × 10^8^ NBs/mL. Figure 3b shows the percentage of cells that were positive for the presence of fluorescent nanoprojectiles. A clear increase in the percentage of cells positive for nanoprojectiles was observed for the two highest laser fluences (1× and 1.5× VB threshold), while for the lowest laser fluence, which is below the VB threshold, this was not the case. At a laser fluence of half the VB threshold fewer VB are formed which are also smaller in size and, therefore, less powerful. It is likely due to this combined effect that hardly any cells are positive for beads below the VB threshold. Upon 5 min incubation with NBs (1.3 × 10^8^ NBs/mL) without laser irradiation, no noticeable fluorescence could be detected in the cells, showing that there is no spontaneous association to or uptake of NBs by cells. As an additional control, cells were incubated for 5 min with pristine IONP core particles (1.3 × 10^8^ NPs/mL) together with an excess of free nanoprojectiles (ratio beads:IONP 300) and irradiated with laser light. In this case a slight increase in the number of cells positive for nanoprojectiles was observed. As it was virtually independent of the applied laser fluence, likely this stems from spontaneous binding and uptake of free nanoprojectiles by cells. Together this shows that (1) VB formation is essential to propel the nanoprojectiles, and (2) fully assembled NBs are needed to induce cell membrane permeabilization and not just a mixture of its components.

Additionally, we investigated to which extent (fragments of) IONP enter cells. For this purpose, inductively coupled plasma-mass spectrometry (ICP-MS) was used to quantify the iron content of cells before and after NB activation. HeLa cells were exposed to 200-NBs and irradiated at the VB threshold fluence. Next, as schematically shown in Fig. 3ci, cells were washed with PBS to remove any remaining extracellular IONP. Following trypsinization of the cells and digestion with aqua regia (3:1 mixture of hydrochloric acid and nitric acid), the iron content was determined using ICP-MS and normalized to the total protein content, as determined by BSA quantification assay (Supplementary Fig. 3), which is a measure for the total number of cells. As a negative control, we included untreated cells, and as a positive control, we included cells with their cell medium after incubation with NBs (i.e., without PBS washing step so that all added NBs are still present). While the positive control showed a much higher Fe content compared to untreated cells, there was no significant increase for the NB-treated cells (Fig. 3cii), showing that IONP fragments do not (substantially) enter cells upon laser activation.

Finally, to confirm that nanoprojectiles from activated NBs effectively cause membrane disruption, we evaluated if a cell-impermeable molecule can enter cells after light-triggered NB treatment. For this, we used propidium iodide (PI) which becomes more fluorescent when entering the cytosol by interacting with intracellular nucleic acids. As a control, cells were irradiated after incubating with IONP core particles (1.3 × 10^8^ NPs/mL) mixed with free nanoprojectiles (1% v/v). As can be seen in a representative confocal image in Fig. 3d, this resulted in only a few cells that became permeable to PI (yellow). This is according to expectation since we already observed before that a simple mixture of uncoupled core particles and projectiles did not cause much penetration of projectiles in cells (cfr. Fig. 3b). In contrast, when cells were treated with intact NBs, the number of PI positive cells increased tremendously. The high magnification image in Fig. 3d shows that cells positive for PI (yellow) also show the presence of fluorescent beads (green; highlighted with white arrows). Vice versa, cells negative for PI had no or very few nanoprojectiles associated with them, clearly establishing the link between PI influx and the presence of nanoprojectiles at the cell membrane.

The experimental data so far suggest that laser activation of NBs above the VB threshold can propel the nanoprojectiles over a distance in space and cause membrane permeabilization in nearby cells. Yet, as explained in the Theory and Simulations section of the Supplementary Information, directed long-range movement of nanoparticles through a viscous medium is far from evident given the large drag force that they experience. Indeed, even if a 200 nm NP is ejected with a substantial initial velocity of 10 m/s, it would come to a halt within a few 10 s of nanometers (Supplementary Movies 1 and 2), even for high density particles (Supplementary Movies 5 and 6). As our numerical simulations in the Supplementary Information show, long distance displacement of NPs requires the presence of a persistent force that carries the particles along. In particular, our simulations show that the force should be present at least until the particle reaches the cell membrane (Supplementary Movies 3 and 7), and even slightly beyond (Supplementary Movies 4 and 8), in order for the particle to effectively penetrate through the cell membrane. As it has been described before that fluid flows can emerge around oscillating and collapsing bubbles in fluids^24^, we therefore hypothesize that long-range displacement of the ejected nanoprojectiles from our nanobombs is enabled by such fluid flows that emerge around the nanobombs upon optical activation and VB formation and which carry along the nanoprojectiles over long distances. It is important to note that the fluid flows by themselves are apparently insufficient to penetrate the cell membrane, since our control experiments with mixtures of core particles and bullets did not lead to PI influx upon laser irradiation (and hence VB formation from the core particles). Therefore, we conclude that the observed membrane penetration is effectively caused by the ballistic impact of ejected nanoprojectiles, which are dragged along in the generated fluid streams around the nanobombs. To understand this better it will be of interest in future work to investigate theoretically and experimentally the nanobomb mechanism in more detail.

### Intracellular delivery in adherent and suspension cells

**Fig. 4:**
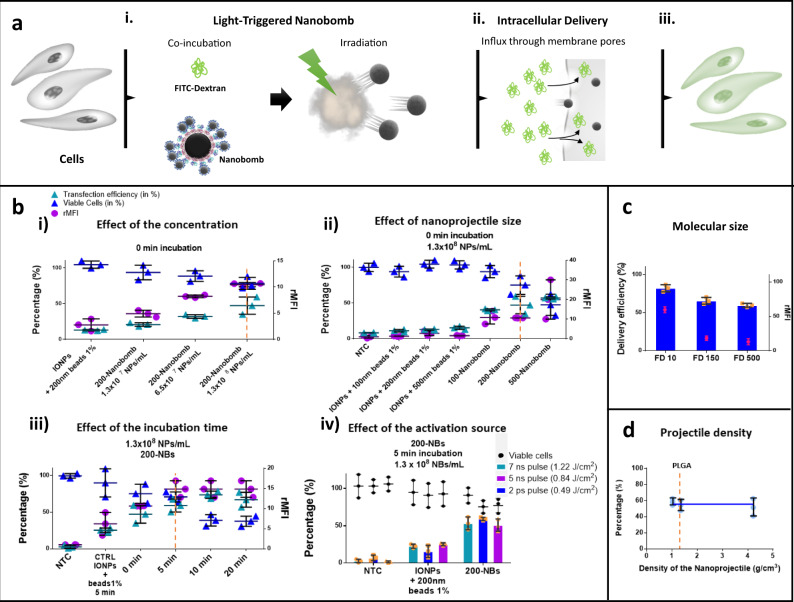
Cytosolic delivery of large macromolecules by laser-activated NBs in adherent HeLa cells. **a** Schematic overview of the direct cytoplasmic delivery mechanism by laser-activated NBs: (i) NBs are co-incubated for a certain period of time with the macromolecules of interest (e.g., FITC-dextran) followed by exposure to one single laser pulse at the VB threshold; (ii) upon laser activation, the nanoprojectiles will puncture the cell membrane of nearby cells, generating transient pores through which the exogenous compounds can enter directly to the cell’s cytosol; (iii) following membrane repair, cells are loaded with the macromolecules. **b** Optimization of the delivery efficiency of FD500 (2 mg/mL) in HeLa cells by 200-NBs with polystyrene beads as nanoprojectiles. Cell viability was determined for all experiments by a CellTiter-Glo assay post-delivery. 200-NBs were activated in every case by irradiation at the VB threshold fluence of 1.22 J/cm^2^ (7 ns pulse at *λ* = 561 nm), unless otherwise specified. Controls were always included of cells incubated with FD500 and a mixture of uncoupled IONP cores at the same concentration as the NBs and polystyrene beads at a 1% v/v excess. (i) Effect of the concentration of 200-NBs when immediately irradiated upon addition to the cell medium together with FD500 (’0 min incubation’); (ii) effect of the nanoprojectile size of 100-NBs, 200-NBs, and 500-NBs and direct irradiation (’0 min incubation’) using a fixed concentration of 1.3 × 10^8^ NBs/mL; (iii) effect of the incubation time of 200-NBs using a concentration of 1.3 × 10^8^ NBs/mL. The vertical orange dashed line indicates the condition selected for further experiments. (iv) Comparison of delivery efficiency and cell viability with different irradiation set-ups. Irradiation was performed at the respective VB threshold fluences. **c** Delivery efficiency of molecular probes of different sizes using 200-NBs: FD10, FD150 and FD500. Effective concentration = 2 mg/mL. **d** Effect of the nanoprojectiles density on the delivery of FD500 using 200-NBs: polystyrene (1.04 g/cm^3^); PLGA (1.3 g/cm^3^); Titania (4.23 g/cm^3^). The vertical orange dashed line highlights results obtained with PLGA NPs as nanoprojectiles, which would be used in further transfection experiments. All results presented correspond to mean ± SD of *n* = 3 biologically independent samples.

Next, we proceeded to evaluate the extent to which NBs are capable of mediating intracellular delivery of large macromolecules. As a model molecule we selected FITC dextran 500 kDa (FD500) for first experiments. Intracellular delivery efficiency was quantified by flow cytometry, while cell viability was determined in parallel using the CellTiter-Glo metabolic assay. Initial experiments were performed on HeLa cells, which is an often-used model cell line for delivery studies. Figure 4a shows a schematic overview of these experiments: (i) cells are co-incubated with cargo molecules (FD500) and NBs for a certain period of time in optiMEM, after which the NBs are activated by scanning of the laser beam over the cell sample; (ii) FD500 will enter into the cytosol after penetration of the nanoprojectiles through the cell membrane; (iii) 5 min later, which should be sufficient for cells to repair the inflicted membrane damage^4^, cells are washed with PBS and supplemented with fresh cell medium for further analysis. Laser activation was performed with the ps irradiation set-up, which is equipped with galvo-mirrors for fast scanning of the laser beam, allowing fast evaluation of several experimental conditions. Starting with 200-NBs, the effect of NB concentration was evaluated. Figure 4bi shows the results in case laser irradiation was immediately applied after adding FD500 and NBs to the cell medium (0 min incubation). Both the percentage of positive cells and the amount of FD500 delivered per cell (rMFI) gradually increased for an increasing NB concentration, while cell viability gradually decreased. At the highest concentration of 1.3 × 10^8^ NBs/mL, 47% FD500 positive cells were obtained for a cell viability of 75%. As a control we used uncoupled IONP core particles at the highest concentration tested, mixed with polystyrene beads (1% v/v). In this case only 13% positive cells were obtained, showing once more that the NB effect is not simply due to the sum of its parts, but requires fully assembled NBs in order to work optimally. Next, for a fixed concentration of 1.3 × 10^8^ NBs/mL, we evaluated the effect of the nanoprojectile size by comparing NBs synthetized with 100 nm (100-NBs), 200 nm (200-NBs) or 500 nm (500-NBs) polystyrene beads (Fig. 4bii). While again virtually no delivery was seen for the controls (IONPs mixed with free polystyrene beads), an increasing trend in delivery efficiency was seen for intact NBs as a function of the nanoprojectile size, which went hand by hand with a decrease in cell viability. However, as cell viability decreased to 47% for 500-NBs, while for 100-NBs and 200-NBs it remained above 75%, we opted to continue with 200-NBs for further experiments in which we tested the influence of NB incubation time (0–20 min) before applying laser irradiation. As shown in Fig. 4biii, both the percentage of positive cells and their rMFI increased by extending the incubation time to 10 min, with no further improvement for 20 min incubation. However, cell viability dropped to below 50% starting from 10 min incubation, so that 5 min incubation was selected as the best condition to continue with. The reason why cell viability decreases with increasing laser fluence is due to more NBs being in close contact with cells or even being endocytosed by cells, leading to gradually more cell damage. Note that for the optimization of these different experimental parameters (NB design, incubation, time, concentration, etc.), delivery efficiency and cell viability was determined 2 h after irradiation. Based on these results, for further experiments on HeLa cells it was decided to continue with 200-NBs at a concentration of 1.3 × 10^8^ NBs/mL, and 5 min incubation time. Next, we confirmed that the delivery efficiency and cell viability does not depend on the laser pulse duration as long as irradiation at the VB threshold is applied (7 ns pulse: VB threshold = 1.22 J/cm^2^, 2 ps pulse: VB threshold = 0.47 J/cm^2^). Since both lasers operate at λ = 561 nm, we additionally performed experiments on a laser irradiation set-up equipped with a 532 nm 5 ns pulsed laser. As this laser set-up was not equipped with dark field imaging (needed to determine the VB threshold), we empirically determined that a laser fluence of 0.84 J/cm^2^ yields similar results as for the other two lasers. The results in Fig. 4biv show equal intracellular delivery efficiency and similar effects on cell viability in all cases, demonstrating the versatility of the NBs with respect to the type of pulsed laser used to activate them.

Next, we evaluated the delivery efficiency as a function of the molecular weight of the cargo molecules. To do this we performed delivery experiments in HeLa cells using the above optimized conditions with FITC-dextran of 10, 150 and 500 kDa (FD10, FD150, FD500) all at the same effective concentration of 2 mg/mL. As shown in Fig. 4c, while the percentage of positive cells only increases slightly for increasing molecular weight, the rMFI decreases more steeply. This can be expected since larger molecules diffuse more slowly, so that fewer of them can enter into the cells while the pores are open.

To measure the time frame that cells need to repair the pores created by NBs, we performed a FD500 delivery experiment by adding the cargo at different timepoints (0, 1, 2, 3, 5, and 7 min) after irradiation of HeLa cells in the presence of 200-NBs (using previously optimized conditions). As shown in Supplementary Fig. 4a, FD500 delivery efficiency decreases as it is added longer after cell membrane permeabilization as a result of membrane resealing. After 3 min, there was no significant difference in FD500 influx any more compared to the non-treated control cells, showing that pores reseal within this timeframe. For traditional photoporation, we reported before that pores formed on HeLa cells reseal in 1 min when using FD500 as a marker^25^. The fact that it is longer for pores generated by NBs could be due to the pores being larger in size, requiring more time to repair.

Next, we proceeded to evaluate the effect of membrane fluidity on the delivery efficiency after NB activation. It is well known that cholesterol plays a crucial role in cell membrane tension regulation and influences mechanical parameters like bending rigidity and elastic modulus^26^. In previous work by Biswas et al., it was demonstrated that biochemical agents like methyl-beta-cyclodextrin (CD) can be used for depleting cholesterol in HeLa cells, leading to a direct increase in the membrane tension, and thus, a decrease in the membrane fluidity^27^. In order to get insight regarding the possible impact of membrane fluidity on pore formation by NBs, we pre-incubated HeLa cells for 1 h with CD at a concentration of 4 mg/mL (determined based on toxicity studies), followed by FD500 delivery with NBs according to the optimized conditions discussed above. The results in Supplementary Fig. 4b show that decreasing membrane fluidity did not influence the effective delivery efficiency, while it did negatively affect cell viability.

Finally, we proceeded to evaluate if nanoprojectile density has any impact on cell membrane permeabilization. For this, we prepared 200-NBs with higher density nanoprojectiles prepared from poly(D,L-lactide-co-glycolide (PLGA, *ρ*_PLGA_ = 1.3 g/cm³) and TiO_2_, *ρ*_TiO2_ = 4.23 g/cm³), which have ~1.3× and 4× higher mass density as compared to polystyrene, respectively. The hydrodynamic size and zeta potential of those NBs and their building blocks are shown in Supplementary Fig. 5a and b. Being a metal-oxide, one may wonder if TiO_2_ NPs themselves may form VBs, similar to the IONP core particles. While this is indeed the case (Supplementary Fig. 5c), their VB threshold (2.58 J/cm^2^) is about 2.5× higher than for the NBs (1.22 J/cm^2^). As indicated by the dashed vertical line, at a fluence of 1.22 J/cm^2^ TiO_2_ NPs do not form VB, so that any observed effects are due to NB activation, and not side-effects due to VB formation from TiO_2_ NPs. When we used those three types of NBs for delivery of FD500 in HeLa cells, no significant difference was found in delivery efficiency (Fig. 4d). Therefore, the here proposed NBs can work independent of the mass density of the nanoprojectiles used, offering great flexibility in their composition. Similar results were obtained by the numerical simulations presented and discussed in the supporting information.

Finally, after evaluation of we verified if light-triggered nanobombs can be used for intracellular delivery in suspension cells as well. For this, we used Jurkat suspension cells, which is an immortalized cell line of human T lymphocytes and a widely used model for hard-to-transfect primary human T cells^22^. Similar to HeLa cells, we delivered FD500 using 200-NBs with polystyrene beads as nanoprojectiles. Keeping the concentration of NBs the same as for HeLa cells (1.3 × 10^8^ NBs/mL), we determined what is the optimal incubation time. As shown in Supplementary Fig. 6a, the delivery efficiency and rMFI increased gradually for longer incubation times, reaching a FD500 delivery efficiency of 49% at 78% viability after 20 min, which again is much better than what is achieved when unassembled IONP and 200 nm beads are used as a control (Supplementary Fig. 6b). We also checked again for this condition to which extent IONP (fragments) may be present in those cells. Supplementary Fig. 6c shows that again a small but non-significant increase was observed for the NB-treated cells, showing that the IONP core particles do not (substantially) enter the cells upon laser activation, similar to HeLa cells (Fig. 3c). Together, these results confirm that the NB system proposed here can be equally applied to suspension or adherent cells, testifying to the flexibility and broad usability of this system.

It is interesting to see that the optimal NB incubation time for HeLa cells is 5 min, while it is 20 min for Jurkat cells. In case of adherent HeLa cells, when NBs are added to the cell medium they will gradually sediment on top of the cells, ensuring good contact with cells. Instead, in case of Jurkat suspension cells the NBs are mixed with the cell suspension which is a more dynamic system which may reduce the probability of NBs associating tightly with the cells. To get a better view on this we prepared NBs with fluorescently labeled nanoprojectiles and measured the level of cell-associated fluorescence as a function of the incubation time. As can be seen in Supplementary Fig. 7, NBs associate indeed more quickly with HeLa’s as compared to Jurkat cells. Upon laser irradiation at the VB threshold, it can be seen for HeLa’s that the nanoprojectiles are associated with the cells already after 5 min, while for Jurkat cells 20 min incubation was needed, in line with the optimal incubation times based on the delivery experiments.

### mRNA and pDNA transfection in adherent and suspension cells

The results so far have shown that light-triggered NBs can be used for intracellular delivery of macromolecular compounds in adherent as well as suspension cells. Encouraged by these results we proceeded to evaluate functional delivery of large nucleic acids, including mRNA and pDNA. In particular we used mRNA (996 nucleotides) and pDNA (5757 base pairs) encoding for enhanced green fluorescent protein (eGFP), always at a concentration of 0.1 μg/μL unless specified otherwise. Rather than using fluorescent polystyrene beads, we made use of 200-NBs with PLGA NPs as nanoprojectiles since PLGA has excellent biocompatibility and biodegradability and, therefore, was considered to be a more relevant system to continue with^28^.

A first question is if NBs may perhaps damage such delicate biological molecules upon activation by laser irradiation? To test this, a mixture was prepared of pDNA (0.1 μg/μL) and NBs (1.3 × 10^8^ NBs/mL) and irradiated with increasing laser fluences. pDNA integrity was subsequently analyzed by gel electrophoresis. As can be seen in Supplementary Fig. 8, no degradation products of pDNA were observed, confirming that even at the highest laser fluences there is no noticeable damage to pDNA. For the highest laser fluences (>0.86 J/cm^2^) it was noted that the bands corresponding to nicked and linear pDNA became fused, which may indicate that VB generation can induce a conformational change of pDNA. However, this had no apparent influence on transfection efficiency.

**Fig. 5:**
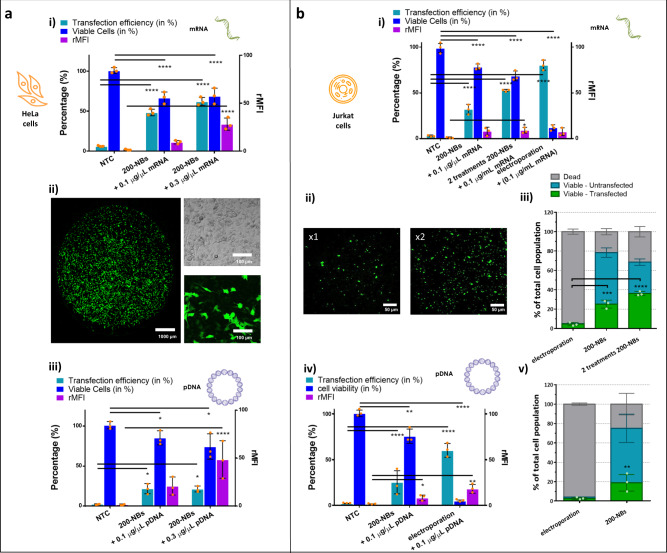
Application of NBs to transfection with mRNA and pDNA of HeLa (adherent) and Jurkat (suspension) cells. **a** Transfection experiments performed on HeLa cells: cells were transfected with eGFP-mRNA and eGFP-pDNA using 200-NBs at 0.1 and 0.3 μg/μL of effective nucleic acid concentration. NBs were added to the cell medium at a concentration of 1.3 × 10^8^ NBs/mL, incubated for 5 min, and irradiated at the VB threshold fluence. The transfection efficiency (i.e., % eGFP+ cells) and expression per cell (rMFI) was determined by flow cytometry 24 h post-transfection. Cell viability was determined in parallel by CellTiter-Glo assay. (i) Results for mRNA transfections. (ii) Representative confocal microscopy images of HeLa cells 24 h after mRNA transfection. (iii) Results for pDNA transfections. **b** Transfection experiments performed on Jurkat cells: experimental conditions were identical to HeLa’s except that the NB incubation time was 20 min. Electroporation experiments were performed using Nucleofection™ according to the manufacturer’s instructions. (i) Results for mRNA transfections. (ii) Representative confocal microscopy images of Jurkat cells 24 h after mRNA transfection, for one (×1) and two consecutive treatments (×2). (iii) The transfection yield calculated for mRNA transfections, showing for each case the percentage of non-viable cells (gray), the percentage of viable but untransfected cells (blue) and the percentage of viable and transfected cells (green). (iv) Results for pDNA transfections and (v) their corresponding transfection yield. All results are represented as mean ± SD for *n* = 3 biologically independent samples. Statistical significance (two-way ANOVA, with multiple comparisons) is indicated when appropriate (*p < 0.05, **p < 0.01, ***p < 0.001, ****p < 0.0001).

Next we proceeded with cell transfections. Conditions were used as optimized for FD500 delivery (1.3 × 10^8^ NBs/mL, 5 min incubation for HeLa cells and 20 min incubation for Jurkat cells). Here we used the laser irradiation set-up with 5 ns laser (fluence of 0.84 J/cm^2^) which is also equipped with a galvo scanning mirror for fast scanning of the laser beam, thus offering higher throughput (10^4^–10^5^ cells/s). The read-out of eGFP expression by flow cytometry or microscopy was performed 24 h after transfection at which point cell viability was measured with the CellTiter-Glo assay as well. Figure 5ai shows the results for the transfection of HeLa cells with eGFP-mRNA. Flow cytometry results indicated 48% eGFP positive cells with a viability of 70%. Moreover, the percentage of transfected cells could be further increased to 61% when using 0.3 μg/μL mRNA, in which case also the rMFI values increased by more than twofold. Figure 5aii shows a large field-of-view confocal image of mRNA transfected cells in a well from a 96 titer plate, as well as a zoomed-in section showing both the transmission and fluorescence channel. Figure 5aiii, finally, shows the results after transfection with pDNA, in which case 22% of the cells were positive for eGFP with a cell viability of 70%. While increasing the pDNA concentration in this case did not translate in an increase in the number of eGFP+ cells, it did result in a more than twofold increase in rMFI.

Performing similar experiments on Jurkat cells revealed that mRNA transfections resulted in 32% eGFP+ cells with a cell viability of 78% (Fig. 5bi). As this means there is still a fairly large fraction of living untransfected cells, we also tried exposing the cells two times to the transfection procedure. This increased the percentage of cells expressing GFP up to 53%, which can be visually appreciated from the confocal images in Fig. 5bii, with only a small decrease of the percentage of viable cells (68%). For comparison we transfected Jurkat cells with mRNA using electroporation as the most explored non-viral transfection method for engineering of hard-to-transfect T-cells^4^. Electroporation of Jurkat cells was performed with a 4D Nucleofector (Lonza) using the protocol recommended by the manufacturer (Pulse code: CL-120; SE cell line solution). As frequently reported^6,29,30^, electroporation resulted in a drastic loss of cell viability with only about 10% of the cells surviving the treatment (measured after 24 h by CellTiter-Glo). Most of the surviving cells were nevertheless transfected (~80%), with a level of expression per cell (rMFI) similar to cells treated by NBs. We summarized these results in Fig. 5biii in which we show the percentage of non-viable cells (gray), the percentage of viable untransfected cells (blue) and the percentage of viable transfected cells (green). The latter is often referred to as the cell transfection yield, and is calculated from multiplying the cell viability (which is calculated relative to the starting amount of cells) with the percentage of transfected cells as determined by flow cytometry (which is gated on living cells). This clearly shows that the mRNA transfection yield for NBs (25%) is markedly higher as compared to electroporation (4%). For the samples that were transfected twice with NBs, the transfection yield even reached 36%. This represents a six-fold increase in transfected cell yield for NBs as compared to electroporation, and a nine-fold increase when the treatment is repeated twice. Note that in this study we asses cell viability making use of an ATP detection assay (i.e., CellTIter-Glo). As it measures metabolic activity it is a more sensitive measure for the health status of a cell instead of a simple live/dead staining which only indicates if a cell is dead or alive. Highly stressed cells may be found to be alive, but can have reduced metabolic activity and, therefore, an altered functionality. This becomes clear in Supplementary Fig. 9, in which we compare the viability determined by live-dead staining using DAPI with the one determined by CellTiter-Glo on Jurkat cells transfected with eGFP-mRNA by electroporation. As can be seen in the figure, after treatment with electroporation, 60% of the cells become positive for GFP expression with a viability of 25% according to live-dead staining and quantification by flow cytometry, similarly as previous reports^31^. Nevertheless, when measuring cell viability based on an ATP metabolic assay (CellTiter-Glo), the observed ‘viability’ was clearly lower. It shows that many of the viable cells according to a simple live/dead stain are actually highly stressed and not very healthy, a finding which has been reported before for electroporated cells^6,32^.

Finally, we also performed pDNA transfections in Jurkat cells with light-triggered NBs (Fig. 5biv), leading to 20% eGFP+ cells with a cell viability of 71%. Electroporation, on the other hand, resulted in 60% eGFP+ cells but with a cell viability of only 6%. The corresponding transfection yield values show that 19% living pDNA transfected cells are obtained with NBs, which is 7.6x more than electroporation (Fig. 5bv).

From a procedural point of view, transfecting cells with laser-activated nanobombs is similar to nanoparticle-sensitized photoporation^9,23,33–36^. In photoporation cells are incubated with photothermal nanoparticles, mostly AuNPs of ~70 nm, which associate with the cell membrane. Upon applying laser irradiation of sufficient intensity, VB are formed from the AuNPs whose mechanical force directly creates pores in the cell membrane. However, so far it has proven challenging for this ‘traditional’ type of photoporation to efficiently transfect cells with large nucleic acids like mRNA and pDNA. To see if our nanobombs form an improvement compared to AuNP sensitized photoporation, we performed comparative photoporation experiments on HeLa and Jurkat cells according to conditions that we optimized and published recently^30^. On HeLa’s, photoporation resulted in only 23% transfected cells for mRNA (Supplementary Fig. 10a), similar to previously published results^30^, which is about half the efficiency of what we obtained with nanobombs. For pDNA photoporation with AuNPs performed even worse, resulting in a negligible amount of only ∼2% eGFP+ cells, while this was about 10x better with nanobombs. On Jurkats mRNA transfections with photoporation resulted in 15% eGFP+ cells (Supplementary Fig. 10b), again only about half the efficiency of nanobombs. For pDNA photoporation only gave 3% eGFP+ cells, while it was 20% with nanobombs. Together this clearly shows the superiority of nanobombs over photoporation with 70 nm AuNPs as the most commonly used photothermal sensitizers.

### Evaluation of spatially resolved mRNA transfections

**Fig. 6:**
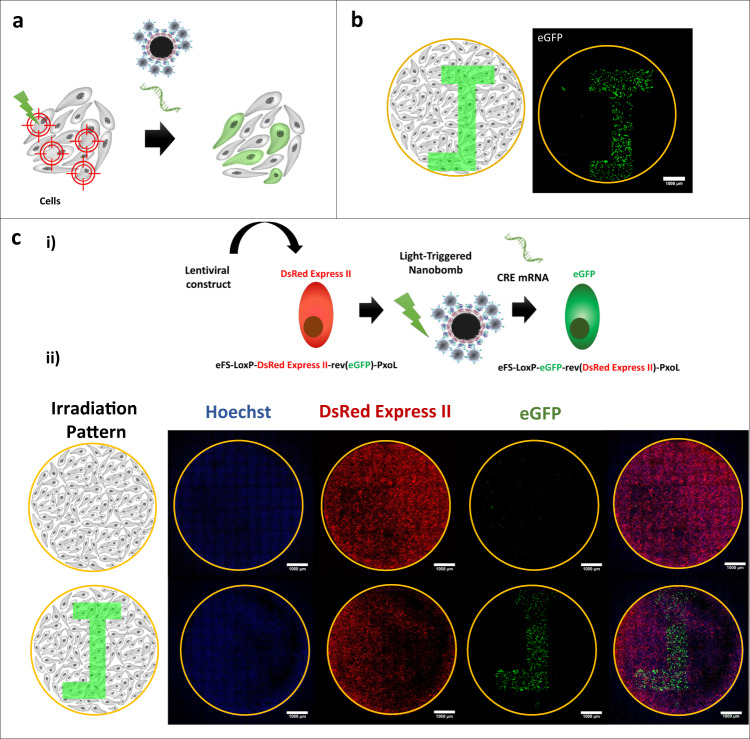
Spatial-selective delivery of mRNA by 200-NBs in adherent cells. **a** Schematic representation of spatial-selective NB-mediated delivery of mRNA. **b** Confocal image 24 h after spatial-selective eGFP-mRNA transfection in HeLa cells according to a “J”-like pattern using 200-NBs at optimized conditions (1.3 × 10^8^ NBs/mL, 5 min incubation, 0.1 μg/μL eGFP mRNA). The bar represents 1000 µm. *n* = 3 images were recorded from three samples. **c** Cell-selective reprogramming with NBs by delivering CRE-mRNA in HeLa cells. (i) HeLa cells were previously transduced with a lentiviral construct containing the eFS-LoxP-DsRed Express II-rev(eGFP)-PxoL cassette. Untransfected cells will only express the red fluorescent protein DsRed Express II whereas in transfected cells with CRE mRNA the DsRed Express II-stop cassette will be inverted between the LoxP sites, resulting in eGFP expression. (ii) Confocal image 72 h after transfection of HeLa cells with CRE-mRNA using 200-NBs. Images were acquired in three spectral channels: Hoechst (nuclear stain, blue), DsRed Express II (red), and eGFP (green). The merged image obtained from the 3 channels it is shown in the last column. First row: control experiment where cells were incubated with mRNA and NBs but without laser irradiation. Second row: CRE mRNA transfection according to the “J”-like pattern. The bar represents 1000 µm. or each condition *n* = 3 images were recorded from three samples.

Since activation of NBs happens by scanning the cell sample with the pulsed laser, it should be possible to transfect cells in a spatially controlled manner by scanning the laser beam according to a pre-defined pattern (Fig. 6a). The confocal image in Fig. 6b shows that cells could indeed be transfected with eGFP-mRNA according to a pattern resembling the letter J (visualized 24 h after treatment). Following this successful proof-of-concept experiment, we proceeded to apply this possibility to a more complex system: cell-selective reprogramming by delivering mRNA encoding for the site-specific recombinase “CRE” in a reporter cell line^37^. For this, HeLa cells were previously transduced with a lentiviral construct containing the eFS-LoxP-DsRed Express II-rev(eGFP)-PxoL. This system presents an easy readout, as untransfected cells will only express the red fluorescent protein DsRed Express II, whereas in cells transfected with CRE mRNA the DsRed Express II-stop cassette will be inverted between the LoxP sites, hence switching on the expression of eGFP (Fig. 6ci). A suitable time point for reading out the conversion of DsRed Express II to eGFP expression was determined to be 72 h after treatment (Supplementary Fig. 11). Spatial-selective cell reprogramming was evaluated by confocal microscopy (Fig. 6cii). Three channels were recorded, being Hoechst (nuclear stain, blue), DsRed Express II (red), and eGFP (green). The first row corresponds to a control where cells are incubated with CRE mRNA (0.1 µg/µL) and NBs but without laser irradiation. As expected, only red staining of the cell’s cytoplasm can be seen corresponding to DsRed Express II expression. Instead, when cells are irradiated in the presence of NBs and CRE-mRNA, expression of CRE recombinase induces eGFP expression according to the “J” pattern. Together these experiments confirm that light-triggered NBs can be used for delivering compounds into cells in a spatially resolved manner by patterned scanning of the laser beam.

## Discussion

The NB concept arises as a nanoscale biolistic solution to deliver large effector molecules into cells for biomedical applications. The self-assembled NB design was tested for activation by laser pulses, which was proven to be able to release the nanoprojectiles from the core upon irradiation with laser pulses at or above the VB threshold fluence (Fig. 2e, f, and Supplementary Fig. 1eiv) independent of the laser pulse duration (2 ps, 5 ns or 7 ns) (Fig. 4biv). We found that ejected nanoprojectiles could penetrate up to 15 µm into a gel matrix (Fig. 2i and Supplementary Fig. 1fi, gi), and more importantly, into the cell’s cytoplasm when the cells are in proximity to the NBs (Fig. 3a, d).

Our experiments have clearly shown that the nanoprojectiles allow to generate local disruptions in the cell membrane through which exogenous compounds present in the external medium can enter in the cell’s cytoplasm, from small molecules like PI (Fig. 3d) to large nucleic acids (mRNA and pDNA), both in adherent and suspension cells (Fig. 5). Our experiments also demonstrated that this membrane disruption effect is linked to the generation of VB by the IONP core, and that the nanoprojectiles must be linked to the core in order to be propelled inside the cells (Fig. 3b). Indeed, when cells are treated with uncoupled core particles and nanoprojectiles, the intracellular delivery efficiency is markedly less (Figs. 4 and 5 and Supplementary Fig. 6).

The process of NB activation can be divided into 2 stages: (1) VB formation, which implies light absorption and heating by the photothermal NP core, and evaporation and expansion of the water surrounding the overheated core; (2) VB collapse, which implies the generation of mechanical effects (e.g., fluid jets and shock waves) which transport the nanoprojectiles over a certain distance in the surrounding (liquid) medium. Due to the high drag force that nanoparticles experience in a viscous fluid (Stokes’ law)^17^, extended directional motion is only possible under the influence of a persistent force. This is confirmed in our simulations (supporting information), which show that an active force must be present until the nanoprojectile has reached the cell membrane because else the projectile quickly comes to a halt due to the large drag force it experiences. This means that likely nanojets and fluid streams must be present upon NB activation which can carry the released nanoprojectiles over a certain distance. The generation of this type of flows after the collapse of nano- and microbubbles near a cell membrane has been already reported by experimental and theoretical studies^38–41^. In such conditions where the released nanoprojectiles are carried along by a fluid flow, their mass (or density) should not play a decisive role on the impact and penetration of a cell membrane, which is indeed what our simulations and experiments (Fig. 4d) pointed at. It will be of interest in future research to investigate theoretically and experimentally how the fluid flows arise around activated nanobombs.

While one may wonder if perhaps ROS generation could play a role in the observed membrane permeabilization, we believe this is rather unlikely. While ROS generation reportedly can induce plasma membrane permeabilization to some extent, only influx of small molecules has been observed so far^42,43^. Instead, in our work we demonstrate successful influx of large nucleic acids like mRNA and pDNA, which requires large pores to be formed in the cell membrane. Together with the observation that NB activation led to deep penetration of nanoprojectiles in a phantom gel upon VB formation, this leads us to conclude that ROS formation – if at all present – is not a major contributor to the observed effects.

It is of interest to compare the results in this manuscript to what has been reported before for pDNA or mRNA transfections of mammalian cells by biolistics or nanoparticle sensitized photoporation (Table 1). Quite variable results have been reported for biolistic pDNA transfections, with incomplete information regarding cell viability or only measured by live/dead staining which usually underestimates cell toxicity as compared to e.g. metabolic viability assays^13^. Limited throughput and transfection efficiency combined with low cell viability is likely the cause why biolistic systems have not been widely accepted for the transfection of mammalian cells. With AuNP-mediated photoporation successful mRNA transfection has been reported, reaching 20% transfection efficiency with >75% viability^30^. pDNA transfection efficiencies of 20–23% have been reported for AuNPs^44^ and carbon black^45^ sensitized photoporation, with viabilities higher than 75%. This is different from what we could achieve for pDNA transfections with gold nanoparticle sensitized photoporation in our control experiments, for which we obtained much lower transfection efficiencies (∼2%). The reason why we cannot replicate those previously reported high transfection efficiencies for gold nanoparticle sensitized photoporation remains elusive to us. Importantly, however, with nanobombs pDNA transfection efficiency could be increased to ~20% on adherent and suspension cells with a cell viability of ~70%. mRNA transfections even outperformed previously published efficiencies by a factor 1.6–2.4. Lastly, it is worth pointing out that in this work we demonstrated that light-triggered NBs outperform electroporation of Jurkat cells by 5.5–7.6 times depending if mRNA or pDNA was used. These promising transfection results, in combination with the fact that NBs can be prepared from biocompatible materials, encourages further studies on the use of activated NBs for safe and efficient engineering of patient-derived immune cells.

**Table 1:** Direct cytosolic delivery of mRNA and pDNA in mammalian cells by nanoparticle-sensitized photoporation and biolistics.

Membrane disruption strategy	Effector molecule	Concentration of effector molecule	Cell type	Efficiency	Viability	Reference
Gene gun biolistic	pDNA	1 μg	HEK 293	∼25%	∼91%^a^	^13^
				∼43%	∼50%	^46^
			MCF7	∼11%	—	
			NIH/3T3	∼2%	—	
Gold nanoparticle-mediated photoporation	mRNA	0.1 μg/μL	HeLa	∼20%	∼80%	^30^
			Jurkat	∼20%	∼75%	
	pDNA	0.1 μg/μL	HeLa	∼2%	∼82%	This work
			Jurkat	∼3%	∼73%	
			MW278	∼23%	∼80%	^44^
Carbon black-mediated photoporation	pDNA	0.02 μg/μL	DU 145	∼22%	∼90%^a^	^45^
NBs	mRNA	0.1 μg/μL	HeLa	∼48%	∼70%	This work
			Jurkat	∼32%	∼78%	
	pDNA		HeLa	∼22%	∼70%	
			Jurkat	∼20%	∼71%	

*mRNA* messenger RNA, *pDNA* plasmid DNA, *NB* nanobomb.

^a^Cell viability assessment by live/dead staining.

Interestingly, we found that NBs maintain the unique possibility of photoporation to transfect cells in a spatial-selective manner (Fig. 6a). We exploited this possibility for selective delivery of mRNA encoding for the site-specific recombinase “CRE”. We showed that it is indeed possible to use NBs to induce cell-selective reprogramming according to a pre-defined spatial pattern. It shows that light-triggered NBs hold the possibility to switch reprogramming factors on or off in a cell-selective spatiotemporal manner, giving control of the cellular diversity and organization. In the future this quite unique feature could allow to generate detailed micropatterns that mimic the zonation of tissues, which is expected to be of benefit for the generation of advanced in vitro engineered tissues.

It is worth highlighting that light-triggered NBs have translational potential as they can be created from materials that are biocompatible and biodegradable. These aspects were already carefully considered in the current NB design. For the VB core we used iron-oxide as a photo-responsive material, which is biocompatible^20^, and can induce light-triggered VB as needed for NB activation^47^. Even though high cytoplasmic concentrations of iron-oxide can induce some toxicity by ROS generation^48^, ICP-MS analysis did not show a significant increase of the iron content in the NB treated cells (Fig. 3c and Supplementary Fig. 6c). With regard to the nanoprojectiles, even though initial characterization experiments were performed with polystyrene beads, we found from theory and experiments that the nanoprojectile’s density is not a determining factor. Therefore, we could select PLGA as a suitable material for the nanoprojectiles, which is a polymer that is already approved by the FDA and EMA due to its high biocompatibility and biodegradability^49^.

Overall, here we reported on a nanotechnological platform, named nanobombs (NBs), for biolistic penetration of cell membranes, enabling fast and efficient intracellular delivery of large macromolecules like mRNA and pDNA. Being composed of biocompatible materials, light-triggered NBs hold potential for clinical translation such as for the generation of engineered cells for cell therapies, including adoptive T cell therapy. On the other hand, the possibility to transfect cells in a spatially resolved manner opens up the road for further research on specific cell differentiation to generate advanced tissue models in tissue engineering.

## Methods

### NB synthesis

NBs consist of two building blocks: a light-responsive core surrounded by nanoprojectiles. 0.5 µm Streptavidin Mono Mag Magnetic Beads (Ocean NanoTech, USA), named ‘IONP’ in the manuscript, were used as core, which consist of a polymeric NP coated with iron oxide, and a final functionalization with a streptavidin monolayer. Different nanoprojectiles were used to form the final NBs: fluorescent polystyrene nanospheres of different sizes (100, 200, and 500 nm) (Invitrogen, USA), 200 nm PLGA NPs (Nanovex Biotechnologies, Spain), and 200 nm TiO_2_ NPs (Microspheres Nanospheres, USA).

Nanoprojectiles were biotinylated using carbodiimide crosslinking of NH_2_-PEG-Biotin to -COOH groups on the nanoprojectiles (Creative PEGWorks, USA). Stable amide bonds were formed after activation with 1-ethyl-3-(3-dimethylaminopropyl)carbodiimide (EDC—Merck, Darmstadt, Germany). When EDC reacts with the carboxyl group of the NPs (nucleophilic reaction), o-Acylisourea is created as an unstable intermediate. This intermediate reacts with the NH_2_-PEG-Biotin, finally forming the biotinylated NPs. To prevent hydrolyzation of the intermediate, *N*-hydroxysuccinimid (NHS—Merck, Darmstadt, Germany) was added. Briefly, a reaction mixture made with 8 mg of EDC, 2.26 mg of NHS and 20 mg of NH_2_-PEG-Biotin, was dissolved in 1 ml HEPES buffered saline (HBS-Buffer). This reaction solution was mixed with 1 ml of carboxylated-NPs and the product was shaken for 24 h at 200 rpm in a HulaMixer® Sample Mixer (Life Technologies, Waltham, USA), protected from light at room temperature. The functionalized NPs were re-dispersed by tip sonication prior to incubation with IONPs (Digital Sonifier S-2500—Branson Ultrasonics, Danbury, USA—20 s with 10 s interval at 15% amplitude).

NBs were synthetized by mixing 0.3 mL of biotin-NPs (excess) with IONPs diluted in PBS to a final volume of 0.5 mL (resulting in an effective NB concentration of 1.3 × 10^9^ NBs/mL). Two different sizes of IONP were evaluated: 0.5 and 1.0 μm. Biotin-NPs and IONPs were incubated for 24 h under rotation to enable self-assembly between the functionalized biotin-beads and the IONPs coated with streptavidin. Next, the non-functionalized beads were removed by 3 consecutive 10 min magnetic washes using the DynaMag™-15 Magnet (Invitrogen, Carlsbad, USA). The obtained NBs were resuspended in 0.5 mL DPBS (Life technologies, Waltham, USA) and stored in the fridge for further use.

For morphological and physicochemical characterization, NBs were washed magnetically and resuspended in ddi water, after which they were transferred either into a disposable folded capillary cell (Malvern, Worcestershire, UK) or into a disposable cuvette (Brand, Wertheim, Germany) for further measurement of their ζ potential or hydrodynamic size, respectively, using a Malvern Zetasizer Nano® (Malvern Instruments Ltd, Worcestershire, UK). The measurements were performed in triplicate at a temperature of 25 °C. In addition, morphological characterization of the synthetized NBs was performed using scanning electron microscopy (SEM). For this, 5 μL of the samples were deposited on a silicon wafer prior to imaging. Scanning electron microscope images were taken with a Zeiss Crossbeam 540 Electron Microscope using a SE2 detector at 1.5 kV.

### VB generation threshold and visualization of NB activation

Different in-house developed optical set-ups were used to determine the laser pulse fluence threshold, which is defined as the laser fluence of a single laser pulse at which 90% of the irradiated NBs generate a VB. In short, NBs (stock: $\sim$1.3 × 10^9^ NBs/mL) were first diluted 100× in PBS and transferred to a 50 mm γ-irradiated glass bottom dish (MatTek Corporation, Ashland, MA, USA). After sedimentation of the NBs, the samples were studied on the different set ups.

#### Set-up with 7 ns laser pulses

Samples were mounted on an inverted microscope (TE2000, Nikon BeLux, Brussels, Belgium) and irradiated with a pulsed laser ($\sim$ 7 ns) tuned at a wavelength of 561 nm (Opolette™ HE 355 LD, OPOTEK Inc., Carlsbad, CA, USA). The laser beam diameter at the sample was 150 µm diameter. The laser pulse energy was monitored using an energy meter (LE, Energy Max-USB/RS sensors, Coherent). An electronic pulse generator (BNC575, Berkeley Nucleonics Corporation), synchronized with an EMCCD camera (Cascade II: 512, Photometrics), was used to trigger individual laser pulses and record dark-field microscopy images before, during and after VB formation. VB can be seen distinctly in dark field microscopy images as brief bright localized flashes of light due to the increase light scattering during their lifetime. By quantifying the number of visible VBs within the laser pulse area (150 µm diameter) for increasing laser pulse fluences, the VB generation threshold can be determined. This set-up was also used for visualization of the nanoprojectile release after irradiation at the VB threshold fluence (1.22 J/cm^2^). For this experiment, 200-NBs were further diluted 1000× in order to have only a few NBs in the irradiation area.

#### 2 ps pulse

The picosecond laser operates with 2 ps pulses (561 nm) generated at 1 kHz pulse repetition frequency using a Ti:Sapphire regenerative amplifier (Spitfire-Ace PM1K, Spectra-Physics, U.S.) seeded by a Ti:Sapphire solid state laser (Mai Tai HP, Spectra-Physics, U.S.) and pumped by a diode-pumped Nd:YLF laser (Ascend 40, Spectra-Physics, U.S.). A homebuilt optical set-up ensured control over the laser fluence at the sample plane by adjusting the pulse energy. Scanning of the laser beam over the substrate was achieved by implementing galvo-mirrors in the optical set-up. Sufficient overlap between neighboring spots was provided to ensure that every location within the scanned area received at least one laser pulse.

Images were analyzed using the ImageJ software (FIJI, https://Fiji.sc/) to visualize and quantify the number of VBs.

### Quantification of nanoprojectile release

NBs were synthetized from 200 nm polystyrene fluorescent beads and 0.5 μm IONPs cores. For a typical experiment 100 μL of NBs (1.3 × 10^8^ NBs/mL) was added to a 0.12 mm deep secure-seal spacer (Invitrogen, California, USA) on a glass sample holder and covered with a coverslip. This sample was mounted on the 7 ns irradiation set-up described above. After irradiation at the VB threshold fluence, the coverslip was removed, and the sample received a magnetic wash to remove (fragments of) the IONP core particles. The fluorescence of the remaining supernatant, which contains the released nanoprojectiles, was quantified using a Victor3 microplate reader (#1420-040, PerkinElmer, Turku, Finland) with excitation at 580 nm and emission at 605 nm. As a positive control, 100 μL of NBs (1.3 × 10^8^ NBs/mL) were incubated with trypsin 10% in PBS for 4 h (assigned as 100% release). Quantification of VB-mediated and enzymatic release was performed according to a calibration curve determined by aliquots of the fluorescent beads of known concentration.

### Projectile penetration in Tissue Phantom Gel

A tissue phantom gel made of 0.5% weight of Agarose (Sigma-Aldrich) was evaluated for NB penetration as it has acoustic and mechanical properties comparable to real tissues^17^. Before solidification, the gel was mixed with 2 mg/mL FD10. In all, 100 μL of this mix was transferred into glass-bottom 96-well plates and allowed to solidify for 1 h. After gelation, 30μL (1.3 × 10^8^ NBs/mL) of far red-fluorescent 200-NBs (0.5 µm IONP core) were added to the gel placed in the 96-well plates. NBs were irradiated with a single laser pulse at the VB threshold after 5 min of incubation time. After irradiation the gel was washed with PBS and 30 μL of PBS was added before imaging. As a negative control, the gel was incubated for 1 h with 30 μL 200-NBs without laser irradiation.

### Cell culture

HeLa cells (cervical adenocarcinoma cells, ATCC® CCL-2™) were cultured in Dulbecco’s modified Eagle’s medium containing growth factor F-12 (DMEM/F-12). Full cell medium was prepared by adding 10% FBS, 2 mM L-Glutamine, and 100 μg/mL penicillin/streptomycin as supplements. Cells were seeded at a density of 10^4^ cells/well in 96-well plates (#92096, TPP, Switzerland) and incubated for 24 h at 37 °C, 5% CO_2_ prior treatment.

Jurkat E6-1 (human leukemic T cells, ATCC® TIB-152™) were cultured in Roswell Park Memorial Institute (RPMI) 1640 medium. Full cell medium was prepared by adding 10% FBS, 2 mM L-Glutamine and 100 µg/mL penicillin/streptomycin as supplements. The cells were maintained in a humidified atmosphere of 5% CO_2_ at 37 °C and the culture medium was renewed every 2 to 4 days. Cells were counted and seeded at a density of 250 × 10^3^ cells/well in 96-well plates (#92096, TPP, Switzerland) and incubated for 24 h at 37 °C, 5% CO_2_ prior treatment.

#### Visualization and quantification of nanoprojectile penetration

After seeding, HeLa cells were washed once with DPBS- and incubated with 200-NBs (1.3 × 10^8^ NBs/mL) for 5 min prior irradiation with different laser fluences on the 7 ns set-up: 0.59 J/cm^2^ (0.5 × VB threshold), 1.22 J/cm^2^ (VB threshold), and 1.77 J/cm^2^ (1.5 × VB threshold). After washing 1× with PBS cells were trypsinized and resuspended in flow buffer prior analysis by flow cytometry.

For visualization of cell penetration, HeLa cells were seeded in 50 mm γ-irradiated glass bottom dishes (MatTek Corporation, Ashland, MA, USA). Cells treated with 200-NBs with “red fluorescent” or “green fluorescent” nanoprojectiles were visualized by confocal microscopy (C1si or C2, Nikon BeLux, Brussels, Belgium) using a ×60 water immersion lens (Plan Apo, NA 1.2, Nikon BeLux, Brussels, Belgium). Cells were first incubated with CellTracker deep red (1000×) for 20 min at 37 °C to stain the cytoplasm, after which they were washed twice with culture medium and incubated with Hoechst33342 (1000×) for 10 min at 37 °C. After staining, the cells were washed with culture medium and imaged using confocal microscopy. Additionally, cells treated with laser-activated NBs were co-incubated with propidium iodide (PI, #P1304MP, Molecular Probes™) to visualize the influx of after bead penetration. Images were analyzed using the NIS-Elements Viewer 4.20 software.

### Flow cytometry

Quantifications based on fluorescence was performed using a CytoFLEX flow cytometer (Beckman Coulter, Suarlée, Belgium). The resulting flow cytometric data were analyzed using the FlowJo (Treestar Inc., Ashland, USA) software.

### ICP-MS measurements

Inductively coupled plasma-mass spectrometry (ICP-MS) was used to evaluate and quantify potential entry of IONP (fragments) into cells. As a negative control, non-treated cells were included, while as a positive control, cells incubated with NBs but without washing steps (i.e. with the full NB dose still present) were used. In every case, the concentration of NBs was 1.3 × 10^8^ NBs/mL for 10000 cells/well seeded, and the incubation time used corresponded to that determined for HeLa and Jurkat cells during the optimization phase (see main text). The cells were collected in a final volume of 100 μL for further digestion as described below.

For ICP-MS analysis, only high-purity reagents were used. Purified water (resistivity 18.2 MΩ cm) was obtained from a Milli-Q Element water purification system (Millipore, France). Pro analysis purity level 14 M HNO_3_ (Chem-Lab, Belgium) further purified by sub-boiling distillation and ultra-pure 9.8 M H_2_O_2_ (Sigma Aldrich, Belgium) were used for sample digestion. Appropriate dilutions of 1 g/L single-element standard solutions (Inorganic Ventures, USA) were used for method development, optimization and calibration purposes. For quantitative element determination, external calibration was relied on as calibration approach (with standards containing 0, 0.5, 1, 2.5 and 5 µg/L Fe), while Ga was used as an internal standard (final concentration: 5 µg/L) to compensate for potential matrix effects and/or signal instability.

The samples were digested via acid digestion in Teflon Savillex beakers, which had been pre-cleaned with HNO_3_ and HCl and subsequently rinsed with Milli-Q water. A mixture of 350 µL of 14 M HNO_3_ and 100 µL of 9.8 M H_2_O_2_ was added to each sample (100 µL) and this mixture was heated at 110 °C on a hot plate for approximately 18 h. Prior to ICP-MS analysis, the digested samples were 10- and 100-fold diluted with Milli-Q water. To avoid contamination, only metal-free tubes were used for standard and sample preparation (15 or 50 mL polypropylene centrifuge tubes, VWR, Belgium). The complete sample preparation procedure, including digestion and dilution of the digests obtained, was carried out in a class-10 clean room (PicoTrace, Germany).

(Ultra-)trace element determination of Fe was carried out using an Agilent 8800 ICP-MS instrument (Agilent Technologies, Japan). This instrument is equipped with a tandem mass spectrometry configuration consisting of two quadrupole units (Q1 and Q2) and a collision-reaction cell (CRC) located in-between both quadrupole mass filters (Q1-CRC-Q2) and therefore, the technique is often referred to as ICP-MS/MS^50^. The sample introduction system comprises a concentric nebulizer (400 µL min^−1^) mounted onto a Peltier-cooled (2 °C) Scott-type spray chamber. The “tandem mass spectrometry” or MS/MS mode enables one to deal with spectral overlap in a more straightforward way than with traditional single-quadrupole ICP-MS instrumentation. In this work, the CRC was pressurized with a mixture of NH_3_/He (10% NH_3_ in He) to overcome spectral interferences seriously hampering (ultra-)trace element determination of Fe, e.g., overlap between the signals of polyatomic interferences, such as ^40^ArO^+^ and ^40^CaO^+^, and that of the ^56^Fe^+^ ions (mass-to-charge—*m*/*z*—56 amu). The introduction of 3 mL/min of NH_3_/He allows the conversion of ^56^Fe^+^ into the ^56^Fe(NH_3_)_2_^+^ reaction product ion that can be detected free from spectral interference at a different m/z ratio (90 amu). The combination of double mass selection with Q1 only allowing ions with an m/z ratio = 56 to enter the CRC and the selectivity of the ion-molecule reactions in the CRC assure that only the ^56^Fe(NH_3_)_2_^+^ contributes to the signal intensity at an m/z = 90 amu. This approach is often referred as to mass-shift and relies on the adequate selection of the best suited reaction product ion formed upon reaction between the analyte ion and the reaction gas by using the product ion scanning tool. To correct for instrument instability, signal drift and matrix effects, Ga was used as an internal standard with the signal intensity for ^71^Ga^+^ being monitored on-mass.

### Intracellular delivery of large macromolecules

Optimization of the delivery parameters: NB concentration, nanoprojectile size, and incubation time, was performed using 500 kDa fluorescein isothiocyanate–dextran (FITC-dextran, Sigma-Aldrich, Bornem, Belgium), abbreviated as FD500, with a final concentration of 2 mg/mL per well. These experiments were performed on a home-built set-up with a nanosecond pulsed laser (5 ns pulse duration, 532 nm wavelength). This set-up is equipped with a galvo scanner for fast scanning of the laser beam and allows to test more conditions than the 7 ns set-up in a shorter amount of time. Scanning of a whole well from a 96-well plate takes only 4 s (as compared to 3 min on the 7 ns set-up). The required irradiation fluence for this set-up was obtained empirically by screening different fluences until identical delivery efficiencies were obtained as with the other set-ups (7 ns and 2 ps pulsed lasers).

For delivery experiments, after washing with PBS, cells were supplemented with or resuspended in 100 µL cell medium containing FD500 (2 mg/mL) and NBs (concentration as indicated in the main text). After a specific incubation time, cells were irradiated with one single laser pulse at the previously determined laser fluence. Following the laser treatment, the cells were washed 3 times with PBS and new medium was added. Quantification of the percentage of positive cells was performed by flow cytometry.

Viability was assessed 2 h after treatment using the CellTiter-Glo® luminescent cell viability assay, as recommended by the manufacturer (Promega, Leiden, The Netherlands). Briefly, HeLa and Jurkat cells were supplemented with an equal volume of CellTiter-Glo® reagent for each well, mixed for 5–10 min using an orbital shaker (120 rpm) and transferred to an opaque 96-well plate. After allowing the plate to stabilize for 10 min, the luminescent signal of each well was measured using a Glomax™ luminometer (Promega, Leiden, The Netherlands).

### Cell transfections by activated NBs

The evaluation of the delivery of functional nucleic acids was performed by the delivery of mRNA or pDNA encoding for the expression of green fluorescent protein (GFP). CleanCap (cc) enhanced green fluorescent protein eGFP-mRNA (5’ moU) with Cat. No.: L-7201 was received from TriLink Biotechnologies (San Diego, California, USA) and stored at −80 °C until use. gWIZ eGFP-pDNA (Cat. No.: P040400, Gelantis, San Diego, CA, USA) was amplified in transformed *E. coli* bacteria and isolated from this bacteria suspension using a Qiafilter Plasmid Giga Kit (Qiagen, Venlo, The Netherlands). Concentration was determined on a NanoDrop 2000c (Thermo Fisher Scientific, Rockford, IL, USA) by UV absorption at 260 and 280 nm and adjusted to a final concentration of 1 µg/µL with HEPES buffer (20 mM, pH 7.2).

Experiments were performed in a similar manner as for delivery of FD500, using the optimized parameters of concentration, nanoprojectile size and incubation time. The main difference is that in this experiment the delivery was performed in optiMEM instead of full medium to avoid nucleic acid degradation. For mRNA experiments, to minimize degradation, an additional step was incorporated in which cells were washed and incubated with Opti-MEM for 10 min^30^. Briefly, after washing with PBS the seeded cells, they were supplemented with or resuspended in 100 µL of optiMEM containing the relevant nucleic acids (0.1 µg/µL, unless specified differently) and 200-NBs (1.3 × 10^8^ NBs/mL), an incubated for 5 or 20 min for HeLa or Jurkat cells, respectively. After this incubation time, cells were immediately irradiated at the previously determined laser fluence at the VB threshold. Following laser treatment, cells were washed 3 times with PBS and full medium was added. Cells were allowed to settle for 24 h prior to analysis (GFP expression and cell viability). Images of transfected cells were taken by confocal microscopy (C1-si, Nikon, Japan).

Additional experiments to evaluate the delivery of small nucleic acids (siRNA) were performed in H1299 expressing green fluorescence protein (GFP) cells (H1299 GFP). Experimental details can be found in the supporting information.

### Transfection of Jurkat cells by nucleofection

Jurkat cells were transfected with eGFP-mRNA using a 4D-Nucleofector™ according to the manufacturer’s recommendations with the SE Cell line 4D-Nucleofector kit (V4XC-1032) (Lonza, Breda, The Netherlands). First, 2 × 10^5^ Jurkat cells together with 2 µg eGFP-mRNA were resuspended in 20 µL SE Cell line solution and transferred to a 16-well Nucleocuvette™ strip. Cells were transfected using the pulse program CL-120 and immediately after supplemented with 80 µL pre-heated culture medium. Finally, 50 µL of that cell suspension was transferred to a 96-well plate already containing 150 µL pre-heated culture medium and incubated at 37 °C, 5% CO_2_ for 24 h prior to analysis by confocal microscopy, flow cytometry and CellTiter-Glo viability assay.

### Lentiviral vector generation

293FT human embryonic kidney (HEK) cells (ThermoFisher) were cultured in Dulbecco’s modified Eagle medium containing 10% FBS, 0.1 mM MEM Non-Essential amino acids, 1 mM sodium pyruvate and 6mM L-glutamine. At 90–95% confluency, cells were transfected by means of a polyethylene imine (PEI)-mediated transfection (Polysciences, Eppelheim, Germany) in a 1:1:1:3 ratio of the envelope (pMD2G; Addgene), packaging (pMDLg/pRRE & pRSV-Rev; Addgene) and transgene encoding plasmids (pLV-GIII-eFS-LoxP-DsRed Express II-rev(eGFP)-PxoL; Vectorbuilder), respectively. Plasmid DNA was isolated using the GenElute™ HP Plasmid Maxiprep kit from Sigma-Aldrich (Overijse, Belgium). At 4 h after transfection, medium was supplemented with 10 µM sodium butyrate (Sigma-Aldrich, Overijse, Belgium) and after 24 h the medium was discarded, and new medium was added. Conditioned medium was collected 48 h and 72 h after transfection. Cell debris was removed by centrifugation at 2500 × *g* for 10 min and 4 °C followed by filtration through a 0.45 µm pore-sized filter. The lentiviral particles were concentrated by ultracentrifugation (Optima L-90K ultracentrifuge; Beckman Coulter) for 90 min at 90,000 × *g* and 4 °C. The pelleted lentiviral particles were suspended in OptiMEM and stored at −80 °C.

### Evaluation of CRE mRNA recombination in CRE-inducible HeLa cells

HeLa cells were cultured in Eagle’s Minimum Essential Medium supplemented 2 mM L-glutamine, 0.1 mM MEM Non-Essential amino acids, 10% FBS, 50 µg/mL streptomycin and 7.33 IU/mL penicillin. At 80–90% confluency, cells were split in a 1:4–1:5 ratio. HeLa cells were cultured in a 24-well format till 80% confluency and transduced at an MOI of 1 with the lentiviral vector carrying the transgene construct: eFS-LoxP-DsRed Express II-rev(eGFP)-PxoL. After 72 h DsRed Express II+ HeLa cells were sorted using a fluorescence activated cell sorter (BD FACSAria cell sorter; Becton Dickinson). 5.106 DsRed Express II+ HeLa cells were electroporated (GenePulser Xcell (Bio-Rad); Square wave, 500 V, 5 ms) in presence of 10 µg CRE mRNA (eTheRNA; Jette, Belgium), inducing an inversion of the floxed sequence. Recombination efficiency of DsRed Express II+ towards eGFP+ cells was monitored for *X* consecutive days via flow cytometry (Attune Acoustic; ThermoFisher Scientific).

### Spatial selective transfections

Here, HeLa cells were irradiated with a 30 µm diameter laser beam according to pattern resembling the letter “J”, at the optimized laser fluence. The selective transfection was first evaluated with eGFP-mRNA (0.1 µg/µL) using 200-NBs as described above for transfection experiments.

Selective transfection was also evaluated in a CRE-inducible HeLa cell line. Information on the generation of this cell line can be found in the Supporting Information. The transduced HeLa cells possess the transgene construct: eFS-LoxP-DsRed Express II-rev(eGFP)-PxoL. The selective delivery of CRE-mRNA (0.1 µg/µL) was performed following the same experimental procedure as described for eGFP-mRNA: irradiation following a “J” pattern, use of 200-NBs according to previously optimized conditions regarding concentration and incubation time. Additionally, the irradiation of a whole well was also evaluated. The read-out point was selected at 72 h to obtain the maximum change in the expression of DsRed to eGFP, as determined by electroporation studies (supporting information). After 72 h cells were analyzed by flow cytometry or by confocal microscopy. For imaging experiments, cells were previously stained with Hoechst33342 (×1000) as described before.

### Statistical analysis

All data are shown as mean ± standard deviation. Statistical differences were analyzed using the Graphpad Prism 8 software (La Jolla, CA, USA). The statistical tests used in each figure are mentioned in the figure caption. Statistical differences with a *p* value <0.05 were considered significant.

### Reporting summary

Further information on research design is available in the Nature Research Reporting Summary linked to this article.

## Supplementary information

Supplementary Information

Description of Additional Supplementary Files

Supplementary Movie 1

Supplementary Movie 2

Supplementary Movie 3

Supplementary Movie 4

Supplementary Movie 5

Supplementary Movie 6

Supplementary Movie 7

Supplementary Movie 8

Reporting Summary

## Data Availability

All data supporting the findings of this study are available within the paper and its Supplementary Information. Any further related information can be provided by the corresponding author upon reasonable request. Source data are provided with this paper.

## Source data

Source Data
