# Publisher Correction: Light triggered nanoscale biolistics for efficient intracellular delivery of functional macromolecules in mammalian cells

**DOI:** 10.1038/s41467-022-30203-z

**Published:** 2022-04-26

**Authors:** Juan C. Fraire, Elnaz Shaabani, Maryam Sharifiaghdam, Matthias Rombaut, Charlotte Hinnekens, Dawei Hua, Jana Ramon, Laurens Raes, Eduardo Bolea-Fernandez, Toon Brans, Frank Vanhaecke, Peter Borghgraef, Chaobo Huang, Félix Sauvage, Tamara Vanhaecke, Joery De Kock, Ranhua Xiong, Stefaan De Smedt, Kevin Braeckmans

**Affiliations:** 1 Laboratory for General Biochemistry and Physical Pharmacy, Faculty of Pharmaceutical Sciences, Ghent University, 9000 Ghent, Belgium; 2 Department of In vitro Toxicology and Dermato-Cosmetology, Faculty of Medicine and Pharmacy, Vrije Universiteit Brussel (VUB), 1090 Brussels, Belgium; 3 Joint Laboratory of Advanced Biomedical Materials (NFU‐UGent), College of Chemical Engineering, Nanjing Forestry University (NFU), 210037 Nanjing, P. R. China; 4 Department of Chemistry, Atomic & Mass Spectrometry – A&MS Research Group, Ghent University, Campus Sterre, Krijgslaan 281-S12, 9000 Ghent, Belgium; 5 VIB Bioimaging Core Ghent, VIB, 9000 Ghent, Belgium

**Keywords:** Biolistics, Transfection, Nanostructures, Nanoparticles, Nanostructures

Correction to: *Nature Communications* 10.1038/s41467-022-29713-7, published online 14 April 2022.

In the original version of the article the images for Figs. 3 and 4 were interchanged, this has now been corrected so that the images and captions for Figs. 3 and 4 match.

