# Supplementary material for: Light triggered nanoscale biolistics for efficient intracellular delivery of functional macromolecules in mammalian cells
Source: Nat Commun. 2022 Apr 14;13:1996. doi: 10.1038/s41467-022-29713-7 (PMC9010410; doi:10.1038/s41467-022-29713-7)
Supplement: Supplementary file 1 — Supplementary Information [file 41467_2022_29713_MOESM1_ESM.docx]

**SUPPORTING INFORMATION**

**LIGHT TRIGGERED NANOSCALE BIOLISTICS FOR EFFICIENT INTRACELLULAR DELIVERY OF FUNCTIONAL MACROMOLECULES IN MAMMALIAN CELLS**

Juan C. Fraire,^1^ Elnaz Shaabani,^1^ Maryam Sharifiaghdam,^1^ Matthias Rombaut,^2^ Charlotte Hinnekens,^1^ Dawei Hua,^1,3^ Jana Ramon,^1^ Laurens Raes,^1^ Eduardo Bolea-Fernandez,^4^ Toon Brans,^1^ Frank Vanhaecke,^4^ Peter Borghgraef,^5^ Chaobo Huang,^3^ Félix Sauvage,^1^ Tamara Vanhaecke,^2^ Joery De Kock,^2^ Ranhua Xiong,^1,3,*^ Stefaan De Smedt,^1,3,*^ Kevin Braeckmans^1,*^

^1^Laboratory for General Biochemistry and Physical Pharmacy, Faculty of Pharmaceutical Sciences, Ghent University, 9000 Ghent, Belgium

^2^Department of *In vitro* Toxicology and Dermato-Cosmetology, Faculty of Medicine and Pharmacy, Vrije Universiteit Brussel (VUB), 1090 Brussels, Belgium.

^3^Joint Laboratory of Advanced Biomedical Materials (NFU‐UGent), College of Chemical Engineering, Nanjing Forestry University (NFU), Nanjing, 210037 P. R. China

^4^Ghent University, Department of Chemistry, Atomic & Mass Spectrometry – A&MS research group, Campus Sterre, Krijgslaan 281-S12, 9000 Ghent, Belgium

^5^VIB Bioimaging Core Ghent, VIB, 9000 Ghent, Belgium

* [Kevin.Braeckmans@ugent.be](mailto:Kevin.Braeckmans@ugent.be), [Stefaan.DeSmedt@ugent.be](mailto:Stefaan.DeSmedt@ugent.be), [ranhua.xiong@njfu.edu.cn](mailto:ranhua.xiong@njfu.edu.cn)

**Theory and simulations**

1. **Displacement of a NP in a viscous medium**

Consider a nanoparticle moving at a velocity $v$ through a viscous medium. This movement will be counteracted by the drag force $F_{d}$:

$F_{d}=-\frac{1}{2}\rho_{m}v^{2}AC_{d}$ (1)

where $\rho_{m}$ is the density of the medium, $A$ is the projected area of the nanoparticle in the direction of movement and $C_{d}$ is the nanoparticle’s drag coefficient which is a function of the Reynold’s number (which in turn depends on the particle’s velocity and shape). Indeed, the Reynold’s number $Re$ for a spherical particle of radius $R$ is:

$Re=\frac{2\rho_{m}vR}{\eta}$ (2)

where $\eta$ is the medium’s dynamic viscosity.

For small Reynolds’ numbers the drag coefficient of a spherical particle is inversely proportional to the Reynold’s number according to:

$C_{d}\approx\frac{24}{Re}=\frac{12\eta}{\rho vR}$ (3)

The drag force then becomes the well-known Stokes drag force:

$F_{d}=-6\pi\eta vR$ (4)

Considering a particle with initial velocity $v_{0}$, its equation of motion under influence of the drag force is:

$m\frac{dv}{dt}=-6\pi\eta vR$ (5)

Integration immediately leads to:

$v=v_{0}e^{- \frac{6\pi\eta R}{m}t}=v_{0}e^{- \frac{t}{\tau}}$ (6)

showing that the velocity will decrease exponentially with decay time:

$\tau=\frac{m}{6\pi\eta R}$ (7)

If we rewrite the mass of the spherical particle as $m=\frac{4}{3}\pi R^{3}\rho_{NP}$, where $\rho_{NP}$ is the nanoparticle’s mass density, then the decay time can be written as:

$\tau=\frac{2R^{2}\rho_{NP}}{9\eta}$ (8)

showing explicitly that the decay will happen more slowly as the particle becomes larger and denser. Since $v=\frac{dr}{dt}$, we obtain the travelled distance by integrating once more, leading to:

$r=v_{0}\tau\left( 1-e^{- \frac{t}{\tau}} \right)$ (9)

In the limit for $t\to+\infty$ we obtain the total distance travelled before the particle comes to a halt:

$r_{tot}=v_{0}\tau$ (11)

So, we see that the total distance travelled is simply proportional to the NP’s initial velocity and its decay time.

Example for 100 nm nanoparticles moving through water**:**

Consider a NP of radius $R=100$ nm with initial velocity $v_{0}=10\frac{m}{s}$ in water at room temperature ($\rho_{m}={10}^{3}\frac{\mathrm{kg}}{m^{3}}$, $\eta={10}^{-3}\frac{\mathrm{kg}}{\mathrm{ms}}$). Then the NP’s initial Reynolds number is $Re=2$, which indeed falls within the case of small Reynolds’ numbers. In case of a polystyrene bead ($\rho_{NP}={1.05\cdot10}^{3}\frac{\mathrm{kg}}{m^{3}}$), the NP’s decay time is $\tau=2.3$ ns and will travel a total distance of only $r_{tot}=23$ nm. For a PLGA ($\rho_{NP}={1.3\cdot10}^{3}\frac{\mathrm{kg}}{m^{3}}$) or TiO_2_ ($\rho_{NP}={4.23\cdot10}^{3}\frac{\mathrm{kg}}{m^{3}}$) NP of the same size this becomes $r_{tot}=28$ nm and 94 nm, respectively.

1. **Numerical simulations of cell membrane penetration by nanoprojectiles.**

As explained above, nanoparticles even at high velocity are expected to come to a halt already after a few 10s of nanometers due to the large drag force that they experience in a liquid. Therefore, some active force must be present that keeps on “carrying along” the nanoparticles over a substantial distance in the cell medium. However, due to the extremely short time scale and small dimensions at which this all happens it is very difficult, if not impossible, to investigate this experimentally. Therefore, we investigated this further by performing numerical simulations of nanoprojectiles carried along by a persistent force towards a cell membrane (Fig. 1a). The viscosity of the fluid on the left hand side of the membrane was set to that of water (0.89 cP), and on the other side it was set to the viscosity of a cell’s cytosol (0.95 cP).^1^ The cell membrane was simulated by a two-phase flow model as previously reported,^2^ using a dynamics mesh modelling with computational fluid dynamics on the ANSYS FLUENT software. Briefly, a spherical object with a diameter ($D$) of 200 nm was set at 10 m/s to move at a defined distance. Afterwards, the velocity was removed, and its moving was simulated by the dynamics mesh in six degrees of freedom model. We considered projectiles with a density ($\rho$) of 1.04 g/cm^3^ (density of polystyrene) or 4.23 g/cm³ (density of TiO_2_ NPs). The particle starts at a position ($L$) 1000 nm to the left of the cell membrane and is moved towards the cell membrane at a constant velocity of 10 m/s, which has been suggested as a lower bound for waterjets that can be formed around cavitation bubbles.^3^ After a certain distance ($l$) the active force is removed, and the remaining motion of the nanoparticle is monitored until it comes to a halt after a time $t_{f}$. Three conditions were examined: (1) the active force stops before the cell membrane ($l=500 \mathrm{nm}<L$), (2) the active force continues until the cell membrane ($l=1000 \mathrm{nm}=L$), (3) and two cases where the active force continues until the particle has passed the cell membrane ($l>L, \mathrm{with} l=1120 nm \mathrm{and} 1500 nm$). Fig. 1b i shows the initial position, the position when the active force is removed, and the final frame of the simulations for a particle of *ρ* = 1.04 g/cm^3^. The upper row corresponds to the first condition ($l=500 \mathrm{nm}$), showing that the nanoparticle indeed stops within a couple of nanometers after the active force is removed, as expected. In the second case, where the active force stops when the particle has reached the cell membrane, a deformation of the cell membrane is observed, but the projectile cannot get through. In the third case, when the active force remains present until 120 nm past the cell membrane, it can be seen that particle disturbs the membrane’s integrity, but still cannot enter the cytoplasm. Only for the fourth case the nanoprojectile passes through and enters the cytoplasmic side (Figure 1b ii). Supplementary Movies 1-4 in the supporting information provide an animated view of those four cases. Simulations performed for particles with a density of ρ = 4.23 g/cm^3^ can be found in the supporting information (Fig. 1 c, Supplementary Movies 5-8), showing that also for higher mass densities the nanoparticle should be actively moved passed the cell membrane in order to get through.


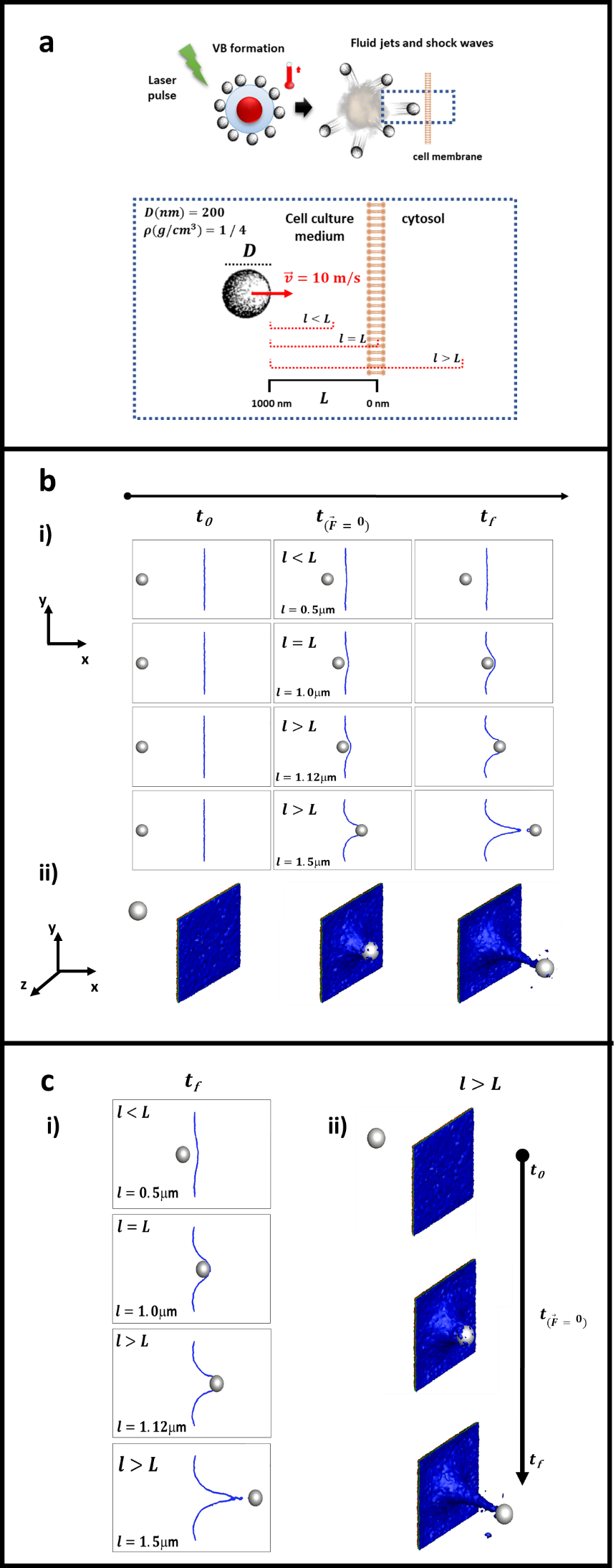


**Simulations of cell membrane penetration by a nanoprojectiles. a)** **(i)**: Schematic overview of theoretical simulations on the propulsion of a nanoprojectile and its penetration of a cell membrane. The nanoparticle had a diameter $D=$ 200 nm and a density $\rho= 1.04$ g/cm^3^ or $4.23$ g/cm^3^, positioned at an initial distance $L=1000$ nm from the cell membrane. Starting from $t_{0}$ the particle is moved with a constant velocity $v=10$ m/s over a distance $l$, after which the active force is removed, and the system is allowed to evolve until the nanoprojectile comes to a halt. b**)** **(i)**: The simulated system ($\rho= 1.04$ g/cm^3^) is shown for $l=500$ nm (top row), $l=L=1000$ nm (middle row), next $l=1120$ nm, and $l=1500$ nm (bottom row). Frames from the simulation are shown for the initial condition, the time point at which the active force is removed, and the final time point when the nanoprojectile has stopped moving. **(ii)**: 3D views of the simulations for $l$ = 1500 nm for the same time points. **c) (i):** Snapshots of 2D simulations for a particle ($\rho= 4.23$ g/cm^3^) traveling under an active force that keeps it moving at a constant velocity of 10 m/s. The particle starts $L=1000$ nm away from the cell membrane, and after it has traveled a distance $l$, the force is removed after which the particle gradually comes to a halt. **(ii)** Snapshots of 3D simulations for the case $l$ = 1500 nm.

**Additional Figures.**


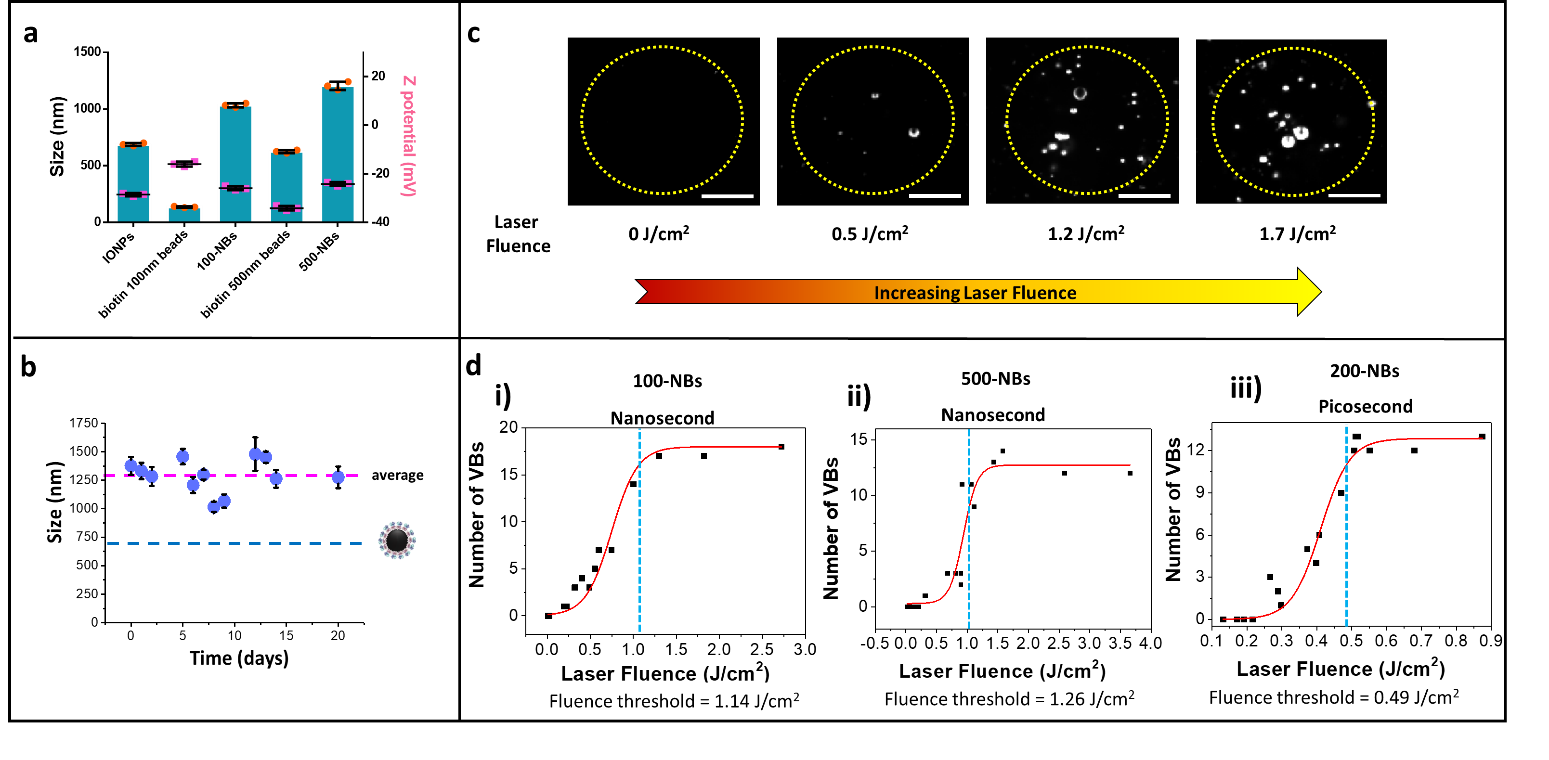


**Figure 1. Synthesis, characterization, and activation of NBs prepared from polystyrene beads of different sizes as nanoprojectiles. a)** DLS and zeta potential characterization of the different building blocks of 100-NBs and 500-NBs (mean ± SD, n=3 independent experiments). **b)** The hydrodynamic size of synthetized 200-NBs was monitored by DLS over a period of 21 days (average size indicated as a dashed pink line). As a reference, the respective core size is indicated by the dashed blue line. It is represented mean ± SD of n=3 independent experiments. **c)** Dark field images showing a colloidal suspension of 200-NBs during VB formation upon irradiation with 1 laser pulse (λ = 561 nm, 7 ns pulse). The irradiation area is indicated by the dashed circle (n=1 independent experiment).the bar represent 50 μm. **d)** Determination of the vapor bubble (VB) fluence threshold for **(i)** 100-NBs and **(ii)** 500-NBs using 7 ns laser pulses, and **(iii)** for 200-NBs using 2 ps laser pulses. n=1 independent experiment. The irradiation wavelength was λ = 561 nm in all cases. The VB threshold is indicated in the graph as a dashed line with the exact value mentioned under the graph.


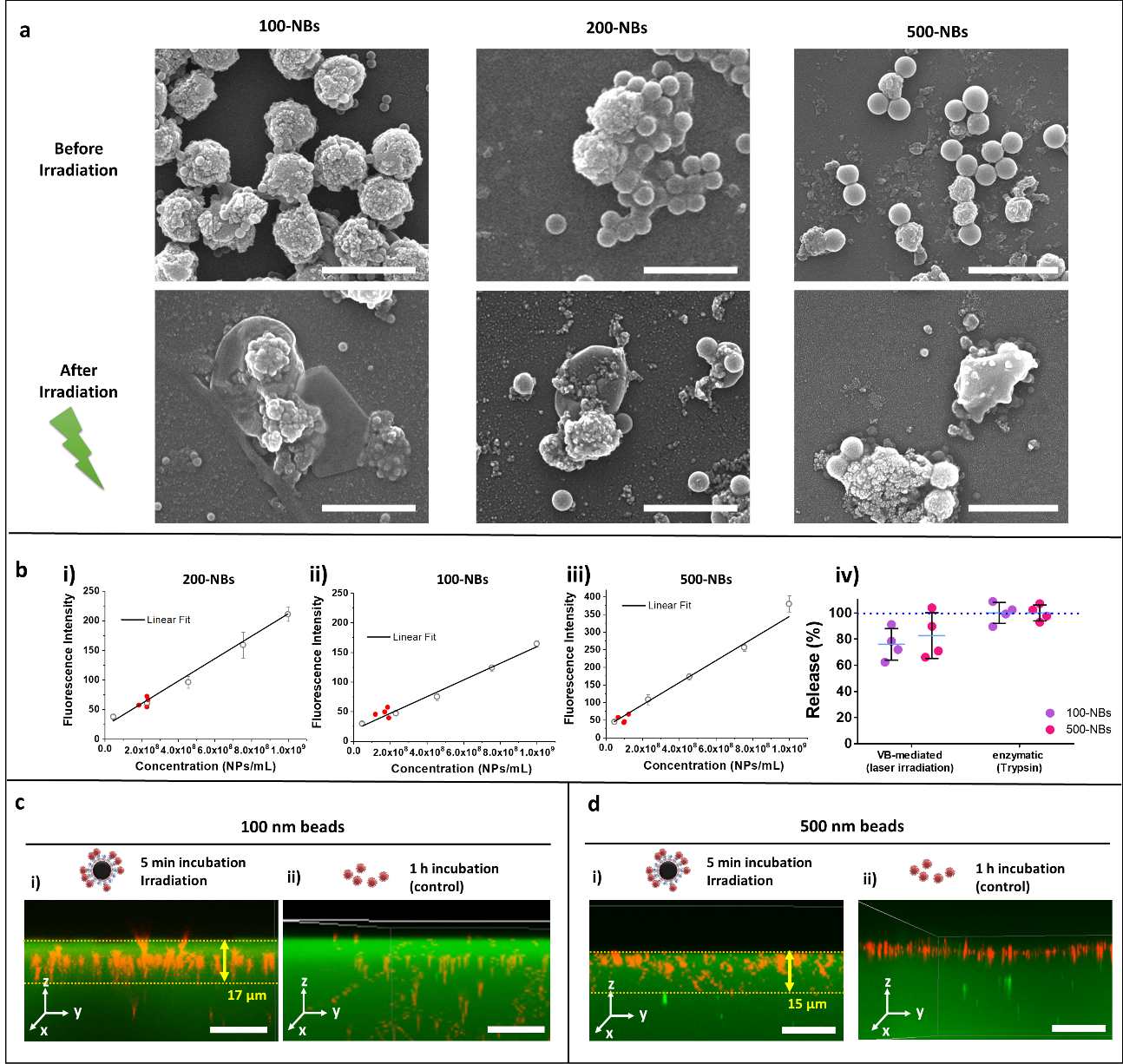


**Figure 2. NB morphology, release, and penetration after activation of NBs prepared from polystyrene beads of different sizes as nanoprojectiles. a)** Representative SEM images of 100-NBs, 200-NBs and 500-NBs before and after irradiation with 1 laser pulse at the VB threshold (for each condition n = 10 images were recorded from two samples.). For 100-NBs and 200-NBs the bar represents 1 μm, and for 500-NBs the bar represents 2 μm. **b)** Quantification of nanoprojectile release. Calibration curve of **(i)** 200 nm, **(ii)** 100 nm and **(iii)** 500 nm fluorescent polystyrene beads (white circles). In red: VB-mediated released beads detected in the supernatant after removal of IONP core particles by magnetic washing (n=4 independent experiments). **(iv)** Quantification of nanoprojectile release. 100-NBs and 500-NBs were activated at the VB threshold fluence (1.14 and 1.26 J/cm^2^, respectively), and release of the fluorescent polystyrene beads was quantified based on the fluorescence of the supernatant after magnetic washing (mean ± SD from n=4 independent experiments). Enzymatic release using 10% trypsin was used as a positive control. Evaluation of nanoprojectile penetration in a phantom gel matrix by **c)** 100-NBs and **d)** 500-NBs. NBs are incubated with a pre-formed gel for 5 min followed by laser irradiation at the VB threshold. As an extra control, 1h incubation only with fluorescent polystyrene beads was included. X-projected 3D confocal image of: **i)** the gel incubated for 5 min with NBs followed by irradiation at the VB threshold (1.3x10^8^ NBs/mL); **ii)** gel incubated for 1h with fluorescent polystyrene beads (1% v/v). For each condition n = 5 images were recorded from two samples. The bar represents 20 μm.


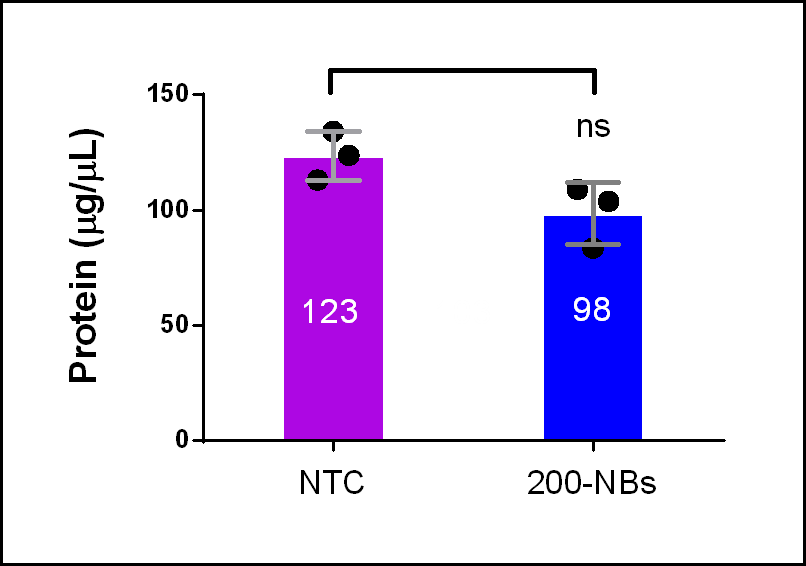


**Figure 3.** Quantification of total protein content of untreated cells (negative control), and cells treated by laser-activated 200-NBs (mean ± SD from n=3 biologically independent samples, t test).


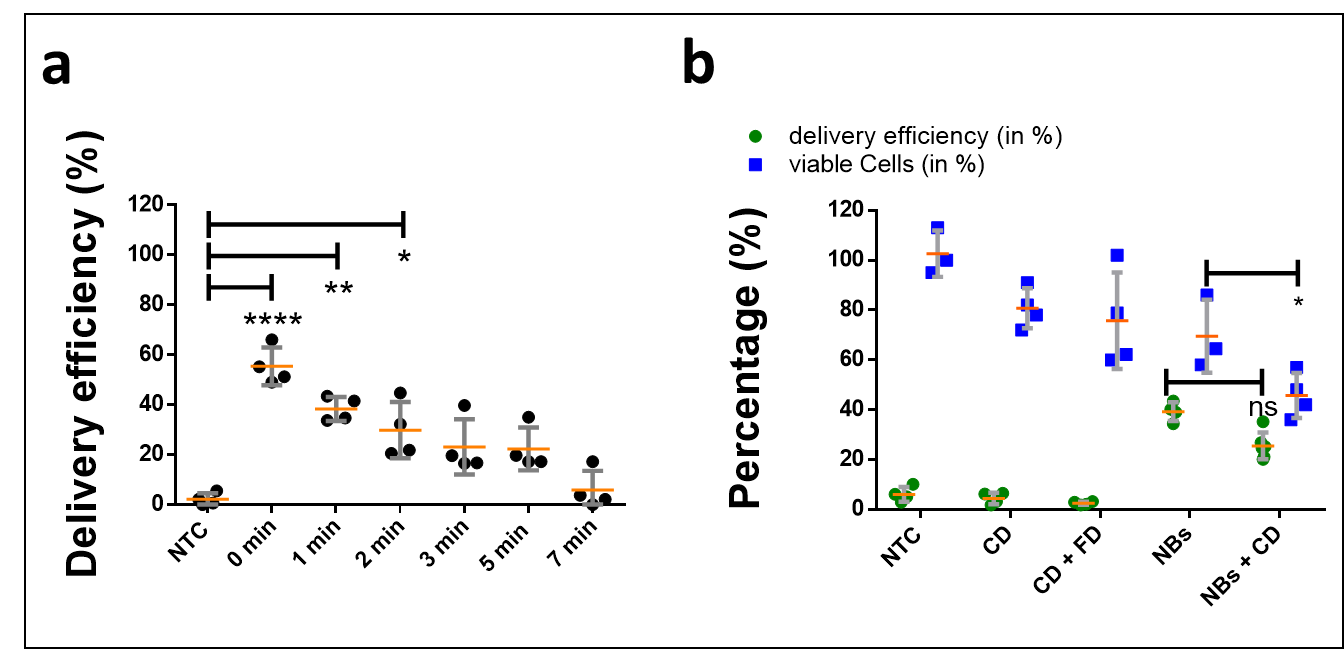


**Figure 4. Pore resealing time and effect of membrane fluidity in HeLa cells. a)** Delivery efficiency of FD500 (2mg/mL) added at different timepoints after 200-NB’s activation (one-way ANOVA, * P<0.05, ** P>0.01, *** P<0.001, **** P<0.0001). **b)** Effect of 1 h pre-incubation with 4mg/mL methyl-beta-cyclodextrin (CD) on the delivery efficiency of FD500 by 200-NBs (two-way ANOVA, with multiple comparisons, * P<0.05, ** P>0.01, *** P<0.001, **** P<0.0001). All results presented correspond to mean ± SD from n=4 biologically independent samples.


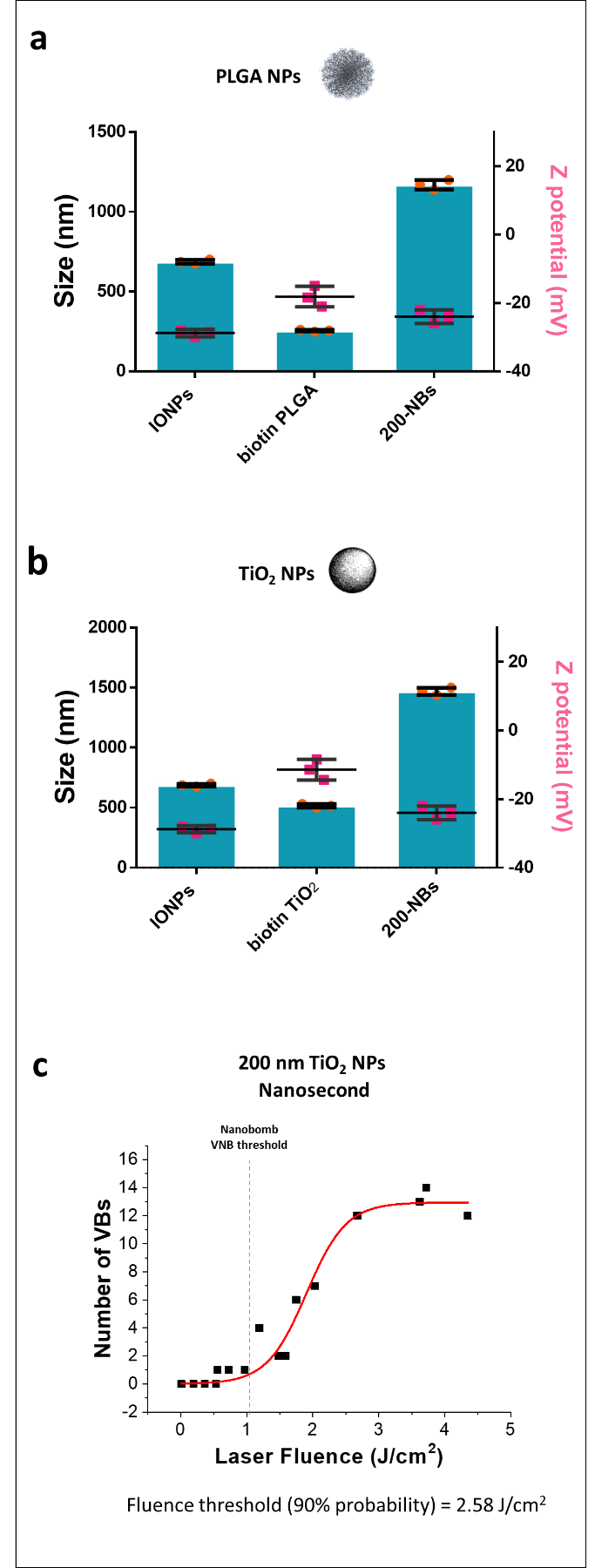


**Figure 5. Synthesis and characterization of NBs using different materials as nanoprojectiles.** DLS and zeta potential characterization of the different building blocks of the synthetized 200-NBs using **a)** 200 nm PLGA NPs or **b)** Titania NPs (mean ± SD from n=3 independent experiments). **c)** Determination of the vapor bubble (VB) fluence threshold for 200 nm Titania NPs using 7 ns laser irradiation (λ = 561 nm). The vertical dashed line indicates the VB threshold of 200-NBs with polystyrene nanoprojectiles as a reference (n=1 independent experiment).


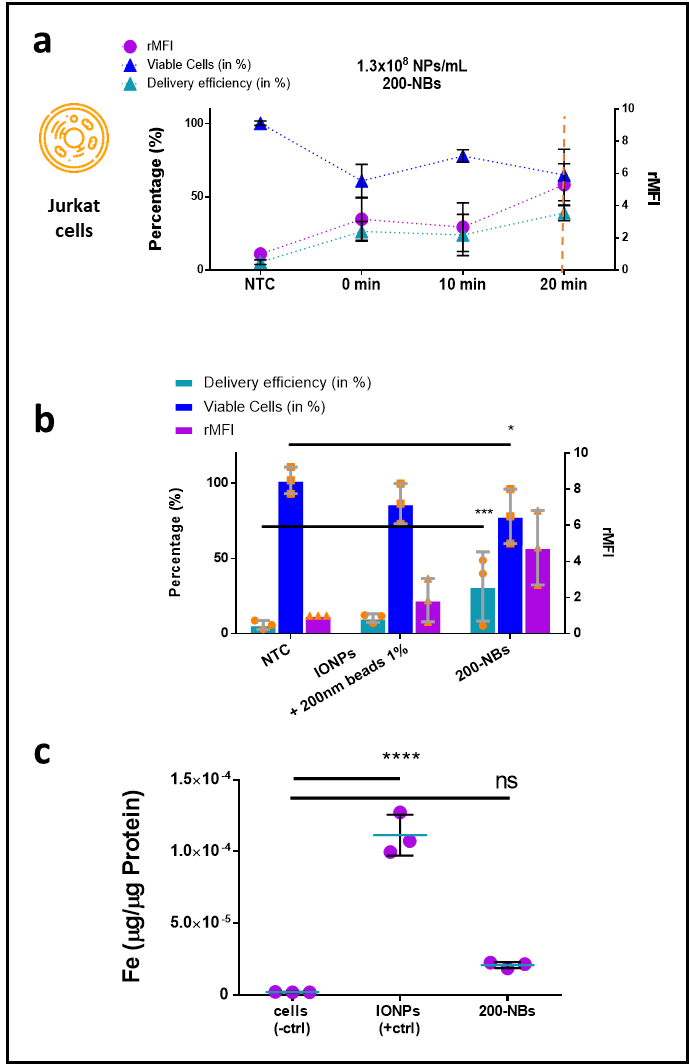


**Figure 6. Optimization of the cytosolic delivery of large molecular probes by activated NBs in Jurkat suspension cells.** **a)** Optimization of the NB incubation time to maximize the delivery efficiency of FD500 (2 mg/mL) in Jurkat cells by 200-NBs with 200 nm polystyrene beads as nanoprojectiles. The concentration of 200-NBs used was 1.3 x10^8^ NBs/mL. The vertical orange dashed line highlights the condition selected for further experiments (20 minutes incubation). Cell viability was determined for all the experiments by a CellTiter-Glo assay 20 min post-delivery. **b)** The delivery efficiency, rMFI and cell viability are shown for the optimized condition (1.3x10^8^ NBs/mL and 20 min incubation) compared to performing the procedure with uncoupled IONP and 200 nm polystyrene beads. (two-way ANOVA, with multiple comparisons, * P<0.05, ** P>0.01, *** P<0.001, **** P<0.0001). All results presented correspond to at least 3 biological replicates. **c)** Quantification of iron content in Jurkat cells by ICP-MS after 200-NB activation. The iron concentration was determined in untreated cells (negative control), cells + cell medium incubated with NBs without any washing (positive control), and cells treated by activated 200-NBs. The results show the Fe mass normalized per μg of protein (one-way ANOVA, **** P<0.0001). The total amount of protein was determined by a BSA protein quantification assay. All results presented correspond to mean ± SD from n=3 biologically independent samples.


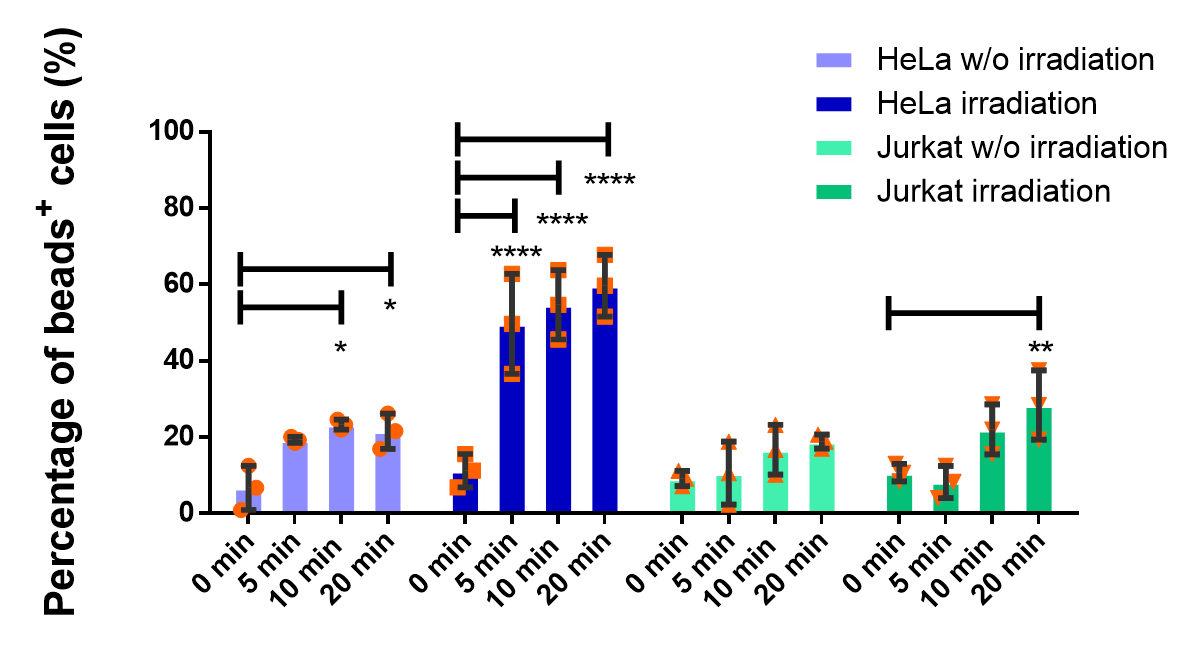


**Figure 7. Interaction of NBs with adherent and suspension cells before and after laser activation.** Flow cytometric quantification over different time points (0, 5, 10, and 20 min) of Hela or Jurkat cells positive for fluorescent nanoprojectiles before and after laser activation of 200-NBs (1.3x10^8^ NBs/mL). Samples were irradiated with a laser pulse fluence at the VB threshold. (two-way ANOVA, with multiple comparisons, * P<0.05, ** P>0.01, *** P<0.001, **** P<0.0001). All results presented correspond to mean ± SD from n=3 biologically independent samples.

**
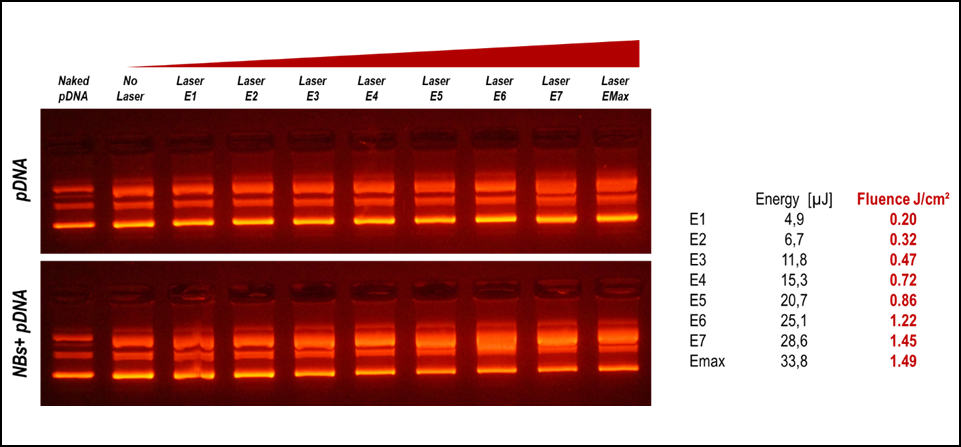
**

**Figure 8. Effect of laser-activated NBs on the integrity of pDNA.** Electrophoretic runs of pDNA 0.1 μg/μL alone (first row) and pDNA 0.1 μg/μL in combination with 200-NBs 1.3x10^8^ NBs/mL (second row) after treatment with laser pulses of increasing fluence (n=1).


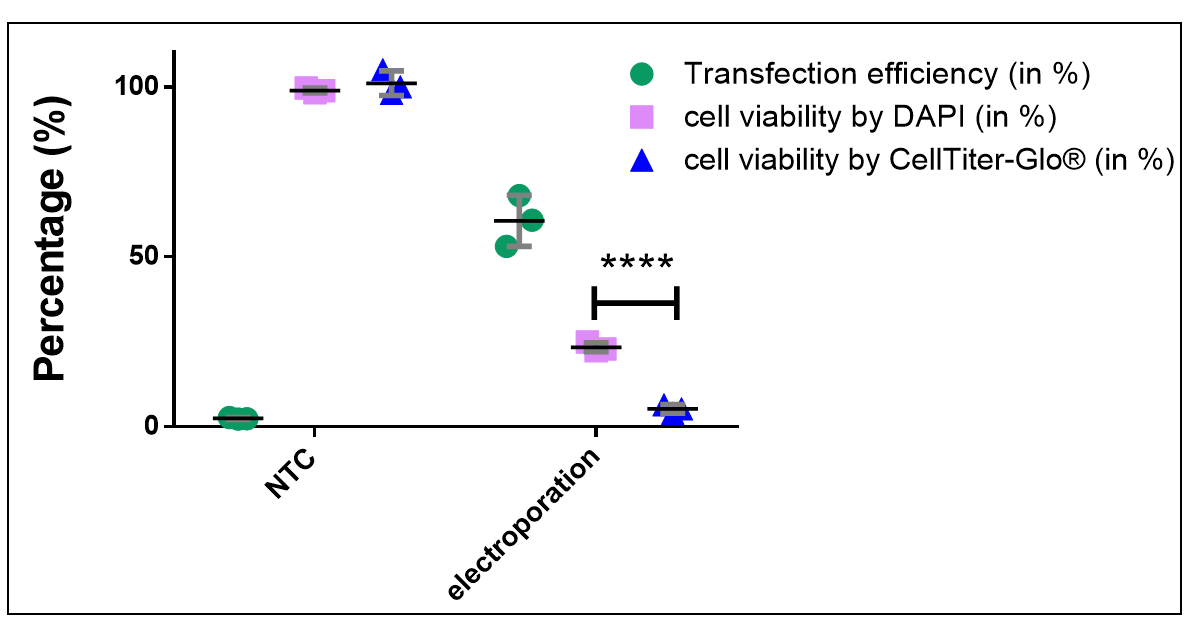


**Figure 9. Comparison of cell viability of electroporated Jurkat cells determined by a metabolic assay and by live/dead staining.** Electroporation experiments were performed using Nucleofection™ according to the manufacturer’s instructions using eGFP mRNA as cargo molecule. Cell viability was determined by CellTiter-Glo assay or based on DAPI staining and flow cytometry analysis. Data are represented as mean ± SD for n=3 biologically independent samples. Statistical significance, from t-test, is indicated when appropriate (****p < 0.0001).


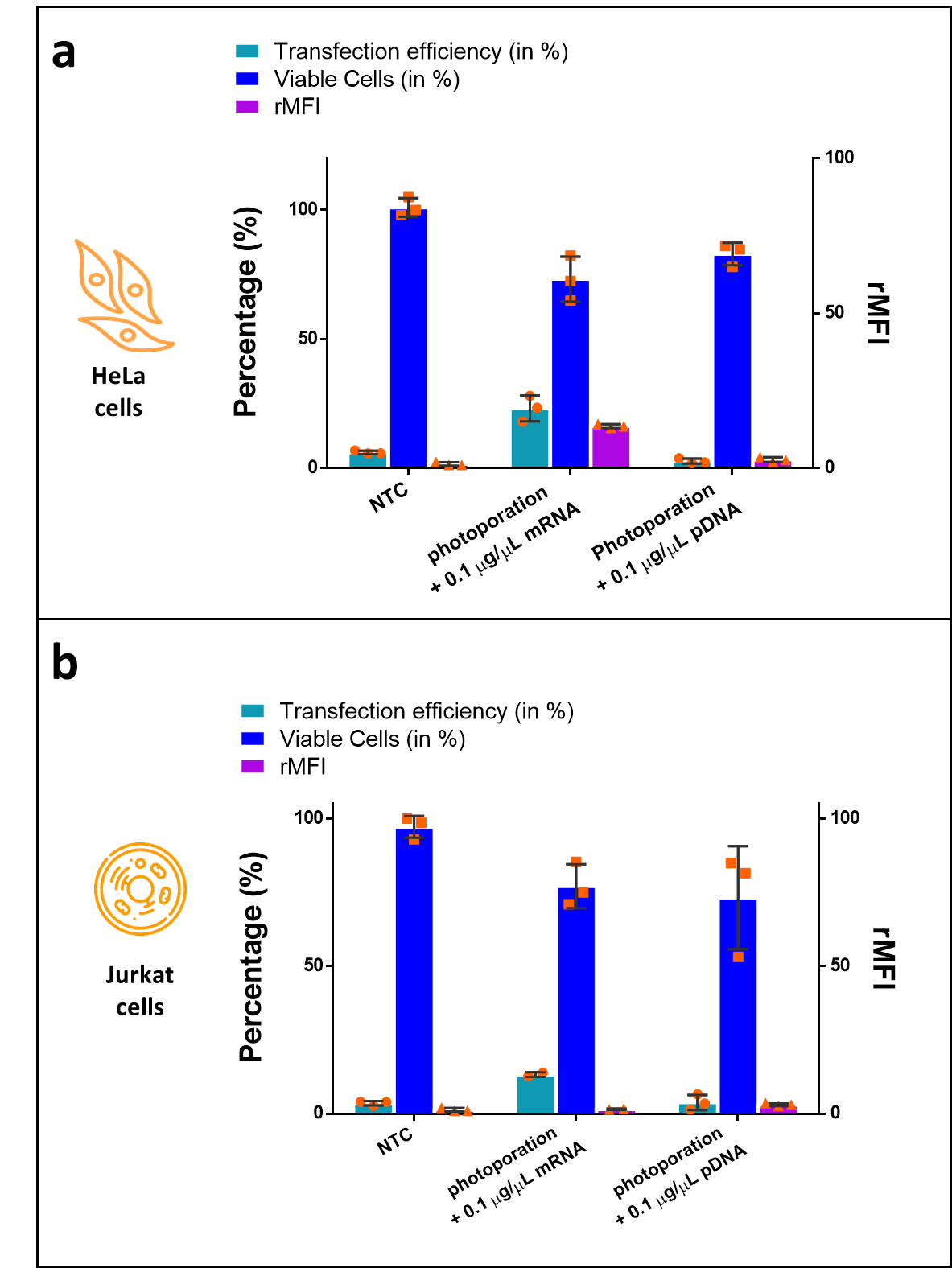


**Figure 10. Photoporation mRNA and pDNA transfections on HeLa (adherent) and Jurkat (suspension) cells.** Photoporation experiments were performed using positively charged 70 nm Au NPs at reported optimal conditions.^4^ Cells were transfected with eGFP-mRNA and eGFP-pDNA at 0.1 μg/μL of effective nucleic acid concentration. The transfection efficiency (i.e. % eGFP+ cells) and expression per cell (rMFI) was determined by flow cytometry 24h post-transfection. Cell viability was determined in parallel by CellTiter-Glo assay. Transfection experiments performed on **a)** HeLa and **b)** Jurkat cells. All results presented correspond to mean ± SD from n=3 biologically independent samples.


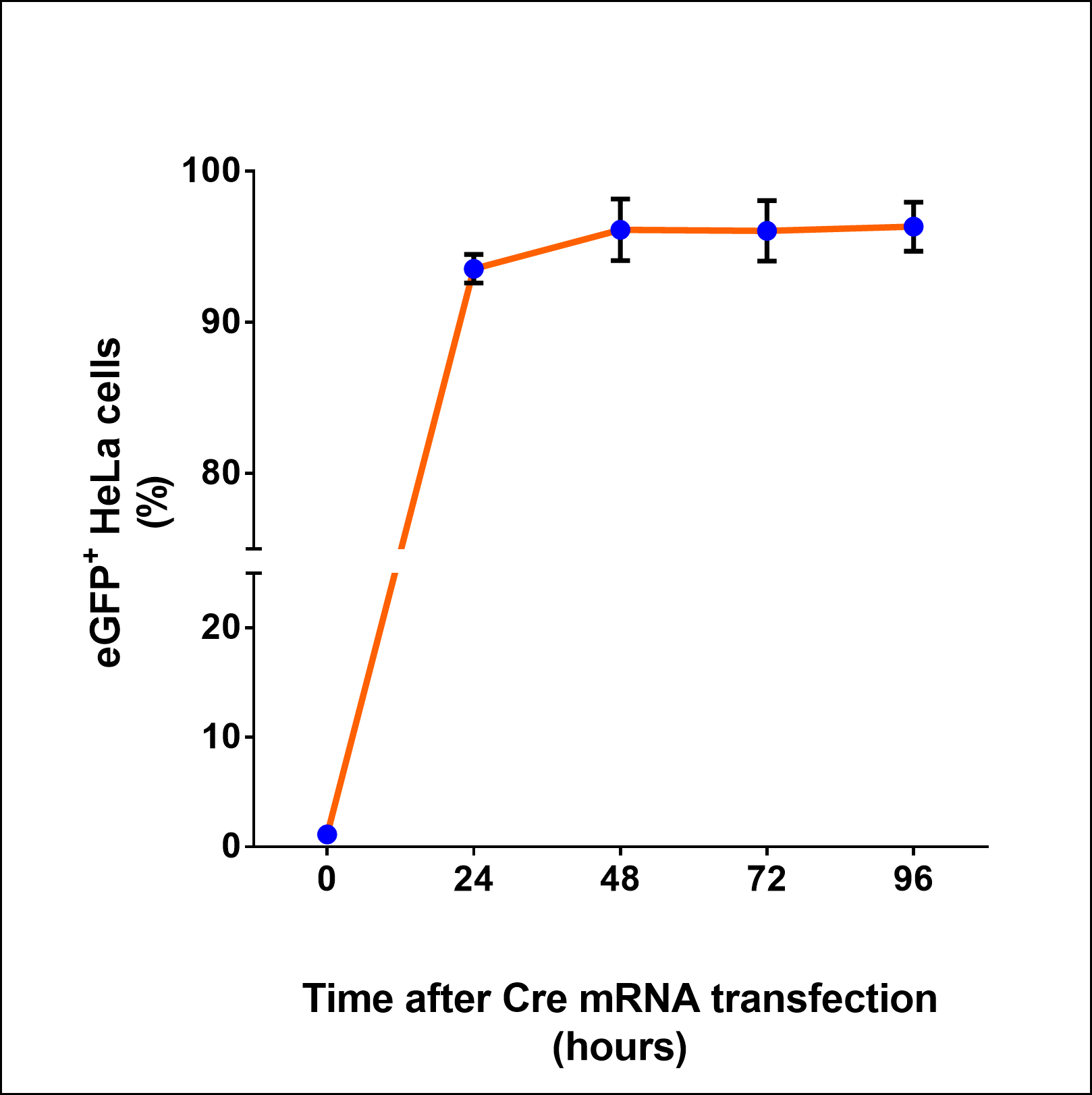


**Figure 11. CRE-mRNA transfection efficiency using electroporation on previously transduced HeLa cells as a function of time.** CRE-mRNA will express a site-specific recombinase that will invert the cassette of previously transduced HeLa cells, switching the expression from DsRed Express II to eGFP. HeLa cells were electroporated (GenePulser Xcell (Bio-Rad); Square wave, 500V, 5ms) in presence of 10µg Cre mRNA/ 5.10^6^ HeLa cells. Mean ± SD represented from n=3 biologically independent samples.

References:

1. Luby-Phelps, K. *et al.* A novel fluorescence ratiometric method confirms the low solvent viscosity of the cytoplasm. *Biophys. J.* **65**, 236–242 (1993).

2. Bruce Stewart, H. & Wendroff, B. Two-phase flow: Models and methods. *J. Comput. Phys.* **56**, 363–409 (1984).

3. Ohl, C. D. *et al.* Sonoporation from jetting cavitation bubbles. *Biophys. J.* **91**, 4285–4295 (2006).

4. Raes, L. *et al.* Intracellular Delivery of mRNA in Adherent and Suspension Cells by Vapor Nanobubble Photoporation. *Nano-Micro Lett.* **12**, (2020).
