# Supplementary material for: Light triggered nanoscale biolistics for efficient intracellular delivery of functional macromolecules in mammalian cells
Source: Nat Commun. 2022 Apr 14;13:1996. doi: 10.1038/s41467-022-29713-7 (PMC9010410; doi:10.1038/s41467-022-29713-7)
Supplement: Supplementary file 2 — Description of Additional Supplementary Files [file 41467_2022_29713_MOESM2_ESM.docx]

**Description of Additional Supplementary Files**

**Supplementary Movie 1.** Modelling with computational fluid dynamics of a spherical object with a diameter (D) of 200 nm with a density (ρ) of 1.04 g/cm3 (density of polystyrene) was set at a position (L) 1000 nm to the left of the cell membrane and is moved towards the cell membrane at a constant velocity of 10 m/s. The active force stops before the cell membrane (l=500 nm <L).

**Supplementary Movie 2.** Modelling with computational fluid dynamics of a spherical object with a diameter (D) of 200 nm with a density (ρ) of 1.04 g/cm3 (density of polystyrene) was set at a position (L) 1000 nm to the left of the cell membrane and is moved towards the cell membrane at a constant velocity of 10 m/s. The active force continues until the cell membrane (l=1000 nm =L).

**Supplementary Movie 3**. Modelling with computational fluid dynamics of a spherical object with a diameter (D) of 200 nm with a density (ρ) of 1.04 g/cm3 (density of polystyrene) was set at a position (L) 1000 nm to the left of the cell membrane and is moved towards the cell membrane at a constant velocity of 10 m/s. The active force continues until the particle has passed the cell membrane (l>L, with l=1120 nm).

**Supplementary Movie 4.** Modelling with computational fluid dynamics of a spherical object with a diameter (D) of 200 nm with a density (ρ) of 1.04 g/cm3 (density of polystyrene) was set at a position (L) 1000 nm to the left of the cell membrane and is moved towards the cell membrane at a constant velocity of 10 m/s. The active force continues until the particle has passed the cell membrane (l>L, with l=1500 nm).

**Supplementary Movie 5.** Modelling with computational fluid dynamics of a spherical object with a diameter (D) of 200 nm with a density (ρ) of 4.23 g/cm³ (density of TiO2 NPs) was set at a position (L) 1000 nm to the left of the cell membrane and is moved towards the cell membrane at a constant velocity of 10 m/s. The active force stops before the cell membrane (l=500 nm L, with l=1120 nm).

**Supplementary Movie 6.** Modelling with computational fluid dynamics of a spherical object with a diameter (D) of 200 nm with a density (ρ) of 4.23 g/cm³ (density of TiO2 NPs) was set at a position (L) 1000 nm to the left of the cell membrane and is moved towards the cell membrane at a constant velocity of 10 m/s. The active force continues until the cell membrane (l=1000 nm =L)

**Supplementary Movie 7.** Modelling with computational fluid dynamics of a spherical object with a diameter (D) of 200 nm with a density (ρ) of 4.23 g/cm³ (density of TiO2 NPs) was set at a position (L) 1000 nm to the left of the cell membrane and is moved towards the cell membrane at a constant velocity of 10 m/s. The active force continues until the particle has passed the cell membrane (l>L, with l=1120 nm).

**Supplementary Movie 8.** Modelling with computational fluid dynamics of a spherical object with a diameter (D) of 200 nm with a density (ρ) of 4.23 g/cm³ (density of TiO2 NPs) was set at a position (L) 1000 nm to the left of the cell membrane and is moved towards the cell membrane at a constant velocity of 10 m/s. The active force continues until the particle has passed the cell membrane (l>L, with l=1500 nm).
